# Preliminary Validation of the Italian Version of the Artificially Intelligent Device Use Acceptance (AIDUA-IT) Scale: Cross-Cultural Adaptation and Psychometric Evaluation

**DOI:** 10.3390/jcm15041578

**Published:** 2026-02-17

**Authors:** Giulia Cavasin, Honoria Ocagli, Dario Gregori

**Affiliations:** 1 Pulmonology Unit, Ca’ Foncello Hospital, Azienda ULSS 2 Marca Trevigiana, 31100 Treviso, Italy; giulia.cavasin.2@studenti.unipd.it; 2 Unit of Biostatistics, Epidemiology and Public Health, Department of Cardiac, Thoracic and Vascular Sciences, University of Padova, Via Loredan 18, 35121 Padova, Italy; honoria.ocagli@unipd.it; 3 Biostatistics & AI for Biomedical Discovery (BIOSTAT-X), Pediatric Research Institute (IRP) “Città della Speranza”, 35127 Padova, Italy

**Keywords:** artificial intelligence, technology acceptance, psychometrics, cross-cultural adaptation, questionnaire validation, AIDUA, structural validity, reliability, healthcare innovation

## Abstract

**Background:** Artificial intelligence (AI) is increasingly integrated into healthcare and public services, making user acceptance a key prerequisite for safe and effective implementation. The Artificially Intelligent Device Use Acceptance (AIDUA) model provides a multidimensional framework for evaluating acceptance of intelligent systems, yet no validated Italian instrument is currently available. **Objectives:** This study aimed to translate, culturally adapt, and preliminarily validate the Italian version of the AIDUA scale (AIDUA-IT) following COnsensus-based Standards for the selection of health Measurement INstruments (COSMIN) and Strengthening the Reporting of Observational Studies in Epidemiology (STROBE) recommendations. **Methods:** A two-phase cross-sectional design was used. Phase one included forward–backward translation, expert review (*n* = 7), and cognitive debriefing (*n* = 8). Phase two assessed structural validity, internal consistency, convergent and discriminant validity, and short-term test–retest reliability in a convenience sample of Italian-speaking adults (*N* = 140), with a subsample completing the test–retest assessment (*n* = 32). **Results:** The hypothesized eight-factor measurement model demonstrated excellent fit (Comparative Fit Index [CFI] = 0.984; Tucker–Lewis Index [TLI] = 0.981; Root Mean Square Error of Approximation [RMSEA] = 0.041; Standardized Root Mean Square Residual [SRMR] = 0.056), with strong standardized loadings (β range: 0.64–0.96) and good internal consistency (Cronbach’s α and McDonald’s ω range: 0.82–0.90). Convergent and discriminant validity were supported, and test–retest reliability was good to excellent across subscales (Intraclass Correlation Coefficient [ICC] range: 0.81–0.90). **Conclusions:** These findings provide initial evidence that the AIDUA-IT is a reliable and valid instrument for assessing acceptance of AI-enabled services in Italy. Further validation in larger and more diverse samples is recommended.

## 1. Introduction

Artificial intelligence (AI) technologies are rapidly transforming healthcare and service industries, offering innovative tools to enhance efficiency, personalization, and user experience. Conversational agents, service robots, and intelligent devices have gained prominence for their capacity to simulate human interaction, support decision-making, and provide guidance in diverse contexts such as education, tourism, and clinical care [1,2].

In healthcare, the integration of AI-based technologies is particularly relevant due to the growing demand for accessible, patient-centered services and the parallel need to optimize clinical resources. From medical service robots assisting in outpatient consultation to AI chatbots supporting patient communication, these technologies promise to improve diagnostic, therapeutic, and organizational processes [3]. However, their successful implementation depends not only on technical performance but also on user acceptance, particularly from patients and end-users directly involved in interactions with such systems [4].

The study of technology acceptance has a long tradition. The Technology Acceptance Model (TAM) and the Unified Theory of Acceptance and Use of Technology (UTAUT) have been widely applied to explain adoption processes, emphasizing constructs such as perceived usefulness, ease of use, and social influence [5,6]. While these frameworks have provided valuable insights, they were originally developed for non-intelligent technologies and often overlook the specific characteristics of AI, such as anthropomorphism, emotions, and the coexistence of acceptance and rejection [7].

To address these limitations, several AI-oriented extensions of classical technology acceptance theories have been proposed in recent years, including the Artificial Intelligence Technology Acceptance Model (AI-TAM) [8]. These models extend technology acceptance theories by incorporating constructs such as trust in AI, perceived intelligence, transparency, and service-related contextual factors to better explain adoption in organizational and commercial setting. However, they mainly focus on cognitive evaluations and contextual determinants, providing limited coverage of users’ emotional responses and perceptions of human-likeness during AI interactions. Gursoy and colleagues [1] developed the Artificially Intelligent Device Use Acceptance (AIDUA) to explicitly address these dimensions. This model conceptualizes acceptance as a three-stage psychological process—primary appraisal, secondary appraisal, and outcome—shaped by six antecedents: social influence, hedonic motivation, anthropomorphism, performance expectancy, effort expectancy, and emotion. The AIDUA model has been applied in various domains, including service industries, chatbots, and healthcare robotics [2,9,10]. Compared with other AI-oriented models, AIDUA explicitly integrates both cognitive determinants and affective components [1]. This theoretical structure may be particularly relevant in healthcare contexts, where emotional responses, perceived empathy, and human-likeness of AI systems can influence trust, engagement, and intention to use [4,9].

In Italy, AI technologies are progressively being introduced across both public services and healthcare. Within the framework of the National Recovery and Resilience Plan (PNRR), the National Agency for Regional Health Services (AGENAS) is implementing an AI infrastructure for primary care and territorial assistance, designed to support clinical decision-making [11]. Similarly, the 2025 Ministry of Health guidelines explicitly call for the integration of AI into national data systems, monitoring frameworks, and governance tools within the healthcare sector [12]. At the operational level, the use of AI in clinical practice is already observable—for instance, a study conducted in Lombardy explored the implementation of AI solutions in regional healthcare organizations [13]. The Istituto Superiore di Sanità (ISS) has emphasized that acceptance is a key determinant of patients’ adherence and long-term engagement, underscoring the need for validated tools to assess user perspectives before large-scale adoption [14]. Despite these initiatives, empirical evidence on how Italian users—including patients, citizens, and healthcare professionals—perceive and accept AI solutions remains limited. No validated instruments are currently available in Italian to capture such perceptions, underscoring the need for a culturally adapted version of the AIDUA questionnaire. This study aimed to translate, adapt and validate the AIDUA questionnaire into Italian, assessing content, face, structural, and construct validity and reliability, according to the Consensus-based Standards for Selection of Health Status Measurement Instruments (COSMIN) framework [15].

## 2. Materials and Methods

### 2.1. Study Design

A cross-sectional psychometric validation study was conducted in two phases.

**Figure 1:**
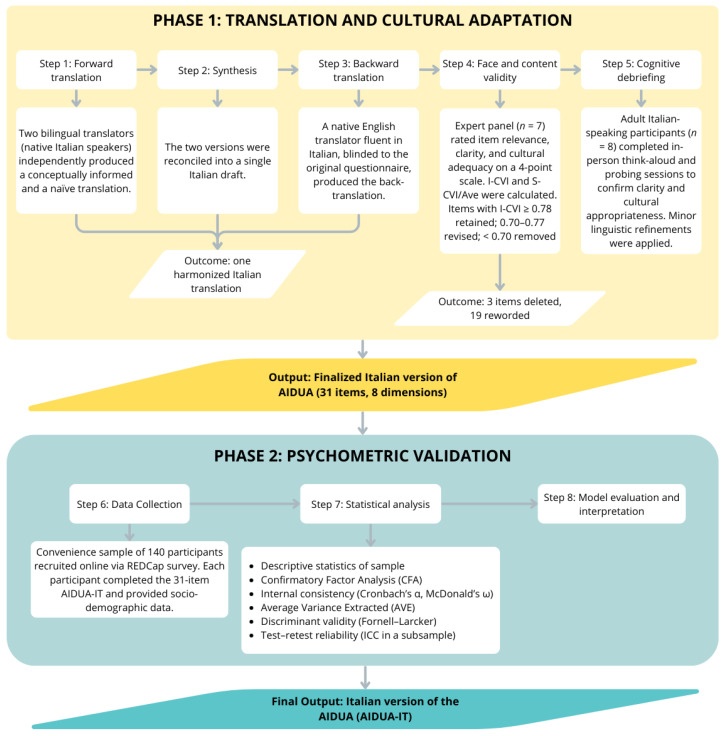
Flow of translation, adaptation and validation phases of the AIDUA-IT questionnaire. Abbreviations: I-CVI = Item-level Content Validity Index, S-CVI/Ave = Scale-level Content Validity Index (Average method), REDCap = Research Electronic Data Capture, AIDUA-IT = Italian version of the Artificially Intelligent Device Use Acceptance (AIDUA) scale, ICC = Intraclass Correlation Coefficient.

The first phase involved the translation and cultural adaptation of the AIDUA, including the assessment of its face and content validity (conducted by a panel of experts) and a pre-testing phase (conducted through cognitive debriefing with eight participants representative of the target population). The second phase aimed to evaluate the psychometric properties of the Italian version (structural validity, internal consistency, convergent and discriminant validity, and test–retest reliability). Figure 1 shows all the steps conducted in this study. This study is reported following Strengthening the Reporting of Observational Studies in Epidemiology (STROBE) recommendations for cross-sectional studies [16].

### 2.2. Participants and Data Collection

The study involved three distinct participant groups. The first group consisted of seven multidisciplinary experts in healthcare, linguistics, psychology, service management and artificial intelligence who in June 2025 assessed the face and content validity.

The second group comprised eight Italian-speaking adults representing diverse ages and educational backgrounds, who participated in July 2025 in individual, in-person sessions, corresponding to the cognitive debriefing phase.

The third group represents the main sample, recruited between August and September 2025 for the psychometric validation. All participants were Italian-speaking adults (≥18 years) who provided informed consent and completed the questionnaire voluntarily. Data were collected through a single online survey administered via Research Electronic Data Capture (REDCap, version 11.1.15; Vanderbilt University, Nashville, TN, USA), hosted at Unit of Biostatistics, Epidemiology and Public Health, University of Padova, a secure, web-based platform designed to support validated data capture for research studies [17,18]. All phases of data collection were completed before the psychometric analyses were conducted. Each construct was measured using the Italian version of the AIDUA questionnaire (AIDUA-IT), consisting of 31 Likert-type items scored from 1 (“strongly disagree”) to 5 (“strongly agree”), except for the Emotion dimension, which employed five-point bipolar adjective pairs (e.g., bored–relaxed). Socio-demographic data were also collected, including age, gender, education, occupational status, and digital familiarity, as recommended [14]. Data comparability was ensured through uniform online administration and identical Italian-language instructions across all participants. The recruitment link to the questionnaire was disseminated via social media platforms (WhatsApp, Instagram, and Facebook) using a snowball sampling approach [19], allowing participants to share the link within their personal and professional networks. As commonly observed in digitally mediated recruitment strategies, this procedure may have favored individuals with higher digital literacy and more frequent use of online technologies, potentially limiting representativeness with respect to less digitally experienced populations [20]. For test–retest reliability assessment, a voluntary subsample of 32 respondents was recruited using the same procedures and inclusion criteria as the main psychometric validation sample and completed the AIDUA-IT twice over a two-week interval.

### 2.3. Original Instrument

The AIDUA questionnaire comprises 34 items measuring eight latent constructs: Social Influence (SI, 6 items), Hedonic Motivation (HM, 5 items), Anthropomorphism (A, 4 items), Performance Expectancy (PE, 4 items), Effort Expectancy (EE, 3 items), Emotion (E, 5 items), Intention/Willingness to Use (I, 3 items), Objection to Use (O, 4 items) [1]. All items are rated on 5-point Likert scales, except Emotion, which uses 5-point bipolar adjective pairs (e.g., bored–relaxed). Formal permission to use and translate the questionnaire was obtained from the original authors on 26 May 2025. The study variables were organized according to the AIDUA theoretical framework, which conceptualizes user acceptance as a multidimensional construct. The outcomes are Intention/Willingness (I) and Objection (O) represented the behavioral outcomes of AI acceptance. Exposures and predictors are represented by the six latent predictors: Social Influence (SI), Hedonic Motivation (HM), Anthropomorphism (A), Performance Expectancy (PE), Effort Expectancy (EE), and Emotion (E). Socio-demographic characteristics (age, gender, education level, professional category) and digital familiarity (hours of use, self-rated computer skills) were treated as contextual variables potentially influencing acceptance scores. No diagnostic or clinical criteria were applicable, as the study population comprised general adult respondents.

### 2.4. Translation and Cultural Adaptation Process

The translation and cultural adaptation of the AIDUA followed established cross-cultural adaptation guidelines, as illustrated in Figure 1 [21,22]. Two bilingual translators, both native Italian speakers, produced independent forward translations (one conceptually informed and one naïve to the construct) which were synthesized into a single draft and back-translated into English by a native English translator blinded to the original version. Face and content validity were then evaluated by the multidisciplinary panel, who independently rated the relevance, clarity and cultural adequacy of each item on a four-point Likert scale, and Content Validity Indices (I-CVI and S-CVI/Ave) were computed [22]. Items with I-CVI ≥ 0.78 were retained, 0.70–0.77 were revised, and <0.70 were considered for deletion. A cognitive debriefing was subsequently conducted with a small sample of eight participants. The main socio-demographic characteristics and digital familiarity of the participants involved in this phase are reported in the Supplementary Materials (File S1, Table S4). Participants were selected to represent heterogeneous age groups, educational levels, and degrees of familiarity with digital technologies. Using a think-aloud and verbal-probing approach, participants paraphrased each item in their own words and provided additional feedback on clarity and cultural appropriateness. Their input informed minor linguistic and semantic adjustments, resulting in the finalized 31-item AIDUA-IT for psychometric validation [23,24]. For example, item 31 describing AI as processing information “in a non-human way” was considered conceptually vague during cognitive debriefing. It was therefore reformulated to explicitly refer to emotional coldness and impersonality, improving semantic clarity.

### 2.5. Statistical Analysis

All items were treated as ordinal variables (Likert-type 1–5). Analyses followed COSMIN and psychometric best-practice guidelines [15].

In order to describe the participants’ characteristics, age was summarized using median and interquartile range (Q1–Q3), and categorical variables were reported as frequencies and percentages. Professional roles were grouped into six macro-categories (front-line, technical, managerial, administrative, student/unemployed, other) based on functional roles and technological exposure, following the UTAUT2 framework [25]. AIDUA model was tested using Confirmatory Factor Analysis (CFA) with a robust Diagonally Weighted Least Squares/Weighted Least Squares Mean and Variance Adjusted (DWLS/WLSMV) estimator, which is recommended for ordered categorical indicators. This approach is particularly appropriate for Likert-type items and moderate sample sizes, as it is based on polychoric correlations and provides more accurate parameter estimates and standard errors under non-normal data distributions [26]. For CFA, cases with missing data on modeled indicators were removed listwise; descriptive statistics and reliability outside the CFA used pairwise deletion where appropriate. Model fit was evaluated using scaled indices: Comparative Fit Index (CFI), Tucker–Lewis Index (TLI), Root Mean Square Error of Approximation (RMSEA) (90% confidence interval [CI], *p*-close) and Standardized Root Mean Square Residual (SRMR). We adopted conventional cut-offs (CFI/TLI ≥ 0.95; RMSEA ≤ 0.06; SRMR ≤ 0.08) [27]. Reliability and validity were assessed through Cronbach’s α, McDonald’s ω, Average Variance Extracted (AVE) and the Fornell–Larcker criterion. Intraclass correlation coefficients (ICC) were calculated using a two-way random-effects model with absolute agreement for single measures [ICC (2,1)], as recommended [28]. For each subscale mean values were computed at Time 1 and Time 2. ICC values were interpreted as poor (<0.50), moderate (0.50–0.75), good (0.75–0.90), and excellent (>0.90) according to the thresholds recommended [28].

Data were analyzed using jamovi (version 2.6.44; The jamovi Project, Sydney, Australia) and Microsoft Excel (version 2024; Microsoft Corporation, Redmond, WA, USA). Jamovi was employed for descriptive, reliability, and confirmatory analyses, while Excel supported data management and preliminary calculations. Statistical significance was set at *p* < 0.05.

### 2.6. Ethical Considerations

The study involved adult volunteers (≥18 years) and did not include patients, clinical data, or any form of intervention. Participation was voluntary and fully anonymous, and no personal data enabling participant identification were collected, in compliance with the European Union (EU) General Data Protection Regulation (GDPR 2016/679). No IP addresses or other potentially identifying metadata were stored, preventing any form of re-identification. Prior to accessing the questionnaire, all respondents provided electronic informed consent, which included information on the study purpose, data protection, and the voluntary nature of participation. In accordance with the Declaration of Helsinki and current Italian and EU regulations on anonymous, non-interventional surveys, ethics committee approval was not required.

## 3. Results

### 3.1. Participant Characteristics

**Figure 2:**
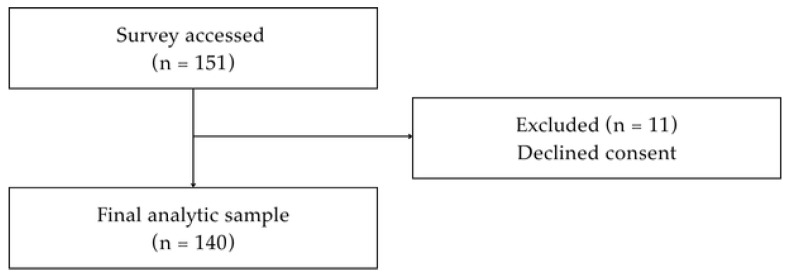
Flowchart of participant recruitment and inclusion in the study.

A total of 151 Italian-speaking adults accessed the survey between August and September 2025. After applying a priori exclusion criteria (absence of informed consent), 11 cases were excluded, yielding a final analytic sample of 140 participants (See Figure 2).

Participants’ ages ranged from 18 to 89 years (Median = 40.5, Q1–Q3 = 27.8–55.0). Most respondents were male (*n* = 88, 63.8%), held at least a high school diploma, and demonstrated good digital literacy with 52.1% (*n* = 73) rating their computer skills as “good” or “excellent”. Regarding professional background, 30.4% (*n* = 42) worked in front-line or operational roles, 14.5% (*n* = 20) in technical or professional areas, 8.7% (*n* = 12) were coordinators or managers, 21.7% (*n* = 30) administrative/clerical staff, 16.7% (*n* = 23) students or trainees, and 8.0% (*n* = 11) other categories (e.g., retirees or freelancers). Table 1 summarizes the characteristics of the sample.

### 3.2. Descriptive Statistics of the AIDUA-IT

Table 2 reports the descriptive statistics for the eight AIDUA-IT subscales. Each score represents the mean of the items belonging to the corresponding latent dimension, providing an overview of score variability to support subsequent psychometric analyses.

### 3.3. Translation and Cultural Adaptation

Expert review (*n* = 7) and cognitive debriefing (*n* = 8) led to the removal of three items and the rewording of nineteen, resulting in a 31-item Italian version of the AIDUA. Items SI2, HM1, and HM5 were eliminated due to low relevance and conceptual redundancy, as indicated by expert ratings (I-CVI < 0.78), consistent with the recommendation to avoid overlapping content and reduce respondent burden in the Italian context [22,23], as presented in Table 3. The retained items ensured comprehensive coverage of each domain based on the AIDUA theoretical framework. Several statements were refined to improve clarity, cultural fit, and linguistic precision. For example, in the social influence domain, “status symbol” was replaced with a neutral phrasing (“reflects a status within my social network”), while hedonic and anthropomorphism items were simplified and clarified (“interacting with AI is engaging”; “AI-based devices have their own will”). Performance and effort items were rephrased with explicit human comparators, and emotional adjective pairs standardized (e.g., “nervous–relaxed,” “sad–happy”). The final 31-item AIDUA-IT maintained conceptual equivalence and cultural appropriateness. All steps of this initial phase are presented, including item-level expert ratings (I-CVI), S-CVI/Ave, and a complete traceability table mapping each original English item to the Italian version with justification of all revisions and eliminations are reported in File S1, along with the AIDUA-IT Final Italian Version.

### 3.4. Construct Validity

The eight-factor AIDUA measurement model examined through CFA showed excellent fit: CFI = 0.984, TLI = 0.981, RMSEA = 0.041 (90% CI 0.027–0.052, *p*-close = 0.902), SRMR = 0.056 (scaled indices; *N* = 138 listwise). All standardized factor loadings were high and statistically significant (β range: 0.64–0.96, all *p* < 0.001). Item reliabilities were strong (R^2^ = 0.41–0.91; Table 4). 

### 3.5. Internal Consistency

All eight subscales showed good internal consistency, with Cronbach’s α and McDonald’s ω between 0.82 and 0.90 (Table 5). According to COSMIN standards, values ≥ 0.70 indicate acceptable reliability, ≥0.80 good, and ≥0.90 excellent [15].

### 3.6. Convergent and Discriminant Validity

Construct validity was examined in accordance with COSMIN guidelines. Convergent validity was supported across all subscales, with AVE values ranging from 0.58 to 0.81, all exceeding the recommended 0.50 threshold [29]. The lowest AVE was observed for Social Influence (0.58), which, although slightly lower than others, remained theoretically acceptable given the conceptual heterogeneity of its indicators (Table 6). Discriminant validity was verified using the Fornell–Larcker criterion. In all cases, the square root of the AVE (√AVE = 0.76–0.90) exceeded the inter-factor correlations (r = −0.58–0.71), and all inter-construct correlations were below 0.85. These results confirm that each latent dimension of the Italian AIDUA scale is empirically distinct while contributing to the overall structure of the model, thus supporting the instrument’s structural validity. All calculation details and supplemental statistical outputs are fully reported (File S3, Tables S8–S10).

### 3.7. Test–Retest Reliability

A subsample of 32 participants completed the questionnaire twice, with a 2-week interval between test and retest administrations. All subscales of the Italian AIDUA showed satisfactory temporal stability across the 2-week interval. ICC (2,1) values ranged from 0.81 to 0.90, indicating good-to-excellent reliability for all latent dimensions (Table 7) [27]. However, the 95% confidence intervals were relatively wide for several subscales, reflecting limited estimation precision due to the small retest sample size (*n* = 32). These results should therefore be interpreted with caution and warrant confirmation in larger independent samples.

## 4. Discussion

This study provides the first cross-cultural adaptation and psychometric evaluation of the AIDUA scale for Italian-speaking users. Overall, the results indicate that the AIDUA-IT demonstrates good structural validity, internal consistency, convergent and discriminant validity and short-term temporal stability, supporting its use for the preliminary assessment of acceptance of AI-enabled services in Italy.

The CFA results showed excellent model fit, with all eight latent constructs loading strongly and consistently on their respective dimensions. This finding replicates the theoretical structure proposed in the original AIDUA model [1] and confirms that the multidimensional nature of AI acceptance holds in the Italian context. The strong loadings observed across constructs such as hedonic motivation, emotion, performance expectancy, and effort expectancy are consistent with the patterns reported in previous applications of AIDUA and related models across different populations [2,9,25]. Importantly, the removal of three items during the adaptation phase did not compromise factorial integrity, indicating that the Italian version preserves the conceptual coverage of the original instrument, in line with recommendations for cross-cultural scale adaptation [21,22]. From a theoretical perspective, the removal of item HM5 (“entertaining”) slightly refines the interpretation of the Hedonic Motivation construct in the Italian context. Whereas the original AIDUA model includes both enjoyment and fun-oriented aspects, the retained items emphasize interest, engagement, and intrinsic involvement in AI interactions. This suggests that, in the Italian sample, hedonic motivation is more closely related to cognitive–affective engagement than to entertainment or playful enjoyment, possibly reflecting cultural differences in how AI technologies are perceived in functional contexts.

Consistently, Hedonic Motivation showed a strong association with Intention to Use (r > 0.60), indicating that affective and experiential factors play a central role in AI acceptance among Italian users. Adoption therefore appears to depend not only on instrumental considerations such as usefulness or efficiency, but also on the extent to which AI interactions are perceived as engaging and emotionally meaningful.

From an applied perspective, this finding has important implications for AI implementation in Italian healthcare or service settings. Systems that are technically accurate but emotionally neutral or impersonal may face resistance. These results highlight the importance of integrating affective design principles and user experience considerations into healthcare AI development. Convergent and discriminant validity findings indicate that the Italian version successfully preserves the conceptual distinctiveness of the eight AIDUA constructs while ensuring that each dimension captures its intended theoretical domain. Rather than simply meeting statistical thresholds, these results suggest that the affective, cognitive, and motivational components underlying AI acceptance remain meaningfully separable in the Italian context. The high internal consistency observed across subscales further reflects the coherence of item content within each domain, supporting the adequacy of the adaptation process. Moreover, the good stability over two weeks indicates that users’ evaluations of AI-related beliefs and attitudes are relatively consistent over short periods, reinforcing the instrument’s suitability for cross-sectional assessments in research and implementation settings.

These results have relevant implications for the Italian context, in which AI-enabled solutions are rapidly expanding in both healthcare and public services. National initiatives such as the AGENAS AI infrastructure program within the PNRR and recent Ministry of Health guidelines highlight the need for robust tools to assess user perspectives prior to large-scale deployment [11]. The availability of a culturally adapted and psychometrically sound instrument offers researchers, developers, and public institutions a standardized way to evaluate user readiness, perceived usability and potential barriers during the design and implementation of intelligent systems. By capturing multiple antecedents and behavioral outcomes, the AIDUA-IT can support user-centered and evidence-informed integration of AI technologies in healthcare and services.

Nonetheless, these findings should be interpreted as preliminary. While the AIDUA-IT shows strong initial performance, further work is required to complete the validation process. Future studies should employ larger and more heterogeneous samples to improve parameter precision, evaluate measurement invariance across demographic and professional groups and test predictive validity in real-world interactions with AI-enabled systems. Longitudinal designs may also clarify how acceptance evolves as exposure to intelligent technologies increases. Such efforts will strengthen the instrument’s applicability and contribute to a more comprehensive, context-sensitive validation program.

Several limitations should be considered. The analytic sample (*N* = 138) resulted in a subject-to-item ratio of approximately 4.5:1, which is below commonly recommended thresholds for confirmatory factor analysis, particularly for complex models with multiple latent dimensions [30,31]. Although this sample size met minimum requirements for a preliminary CFA, larger and more heterogeneous samples are needed to improve the stability and precision of parameter estimates and to support advanced procedures such as multi-group measurement invariance testing. Moreover, the sample showed a male predominance (63.8%), which may have influenced acceptance-related constructs and limits the generalizability of the findings. Previous research indicates that men tend to engage more frequently with generative AI chatbots across a broader spectrum of applications and show greater interest in their relevance to future career prospects, whereas women tend to use such tools primarily for text-related tasks and express greater concerns about critical and independent thinking [32]. Although the present sample size did not allow for reliable multi-group confirmatory factor analysis to formally test measurement invariance across genders, these findings highlight the importance of including gender-balanced samples in future validation studies and formally assessing potential measurement differences across genders. The use of a non-probability, digitally mediated recruitment strategy, likely introduced selection bias by oversampling individuals with higher digital familiarity. This sampling characteristic may have inflated acceptance-related constructs, particularly Effort Expectancy and Performance Expectancy, as digitally skilled participants may perceive AI-enabled systems as easier to use and more useful than the general population (particularly the elderly or clinical populations) [20]. Future studies should incorporate offline recruitment pathways (e.g.,community settings, outpatient clinics, paper surveys) to ensure broader population coverage. In addition, measurement invariance across key subgroups (e.g., age, gender, and professional background) was not examined in the present study. Future research should explicitly test configural, metric, and scalar invariance to ensure that the AIDUA-IT measures the same constructs equivalently across different demographic and professional groups and to support meaningful comparisons between populations. The two-week test–retest reliability analysis was based on a small subsample (*n* = 32), resulting in relatively wide confidence intervals around ICC estimates. This limits the precision of reliability estimates and warrants confirmation in larger samples. Finally, the study relied on self-reported data collected in a single session, which may introduce information bias and common-method variance. Participation was voluntary and anonymous, helping to reduce—but not eliminate—the potential for social desirability effects given the prominence of AI in current public discourse.

## 5. Conclusions

This study offers an initial psychometric evaluation of the AIDUA-IT, demonstrating good structural validity, internal consistency and short-term temporal stability. The results indicate that the instrument is suitable for preliminary assessment of attitudes toward AI-enabled services within the Italian population. Beyond its use in research settings, the AIDUA-IT may serve as a practical tool to support pilot implementations of AI-based systems in Italian healthcare organizations. It can be used to conduct structured needs assessments, identify barriers and facilitators to adoption among healthcare professionals and patients, and inform the design and evaluation of human-centered AI interventions prior to large-scale deployment. However, given the sampling constraints and the absence of predictive or invariance testing, these findings should be interpreted as foundational rather than definitive. Additional validation work is necessary to confirm the instrument’s applicability across different demographic and professional groups and to establish its ability to predict real-world engagement with AI technologies. By providing the first standardized measure of AI acceptance tailored to the Italian context, this study lays important groundwork for future research and supports user-centered strategies for the responsible deployment of intelligent systems in healthcare and public services.

## Figures and Tables

**Table 1:** Characteristics of the sample.

	Overall (*N* = 140)
Age	
Median (Q1–Q3)	40.5 (27.8–55.0)
Range	18.0–89.0
Gender	
missing	2
female	50 (36.2%)
male	88 (63.8%)
Educational level	
middle school	12 (8.6%)
high school diploma	55 (39.3%)
bachelor’s degree	31 (22.1%)
master’s degree	37 (26.4%)
phd or postgraduate specialization	5 (3.6%)
Professional category	
missing	2
front-line/operational	42 (30.4%)
technical/professional	20 (14.5%)
managerial/coordinator	12 (8.7%)
administrative/clerical	30 (21.7%)
student/trainee/unemployed	23 (16.7%)
other/non-classifiable	11 (8.0%)
Computer skills (self-rated)	
poor	9 (6.4%)
fair	15 (10.7%)
sufficient	43 (30.7%)
good	58 (41.4%)
excellent	15 (10.7%)
Main purpose of AI use	
work	73 (52.1%)
leisure	66 (47.1%)
none	1 (0.7%)
Average weekly digital activity	
less than 1 h	3 (2.1%)
1–5 h	19 (13.6%)
5–10 h	25 (17.9%)
10–15 h	23 (16.4%)
more than 15 h	70 (50.0%)

Values are reported as *n* (%). Q1–Q3 = first and third quartile.

**Table 2:** Means, standard deviations (SD), and observed ranges for each subscale, computed as the average of the items within each latent dimension.

	SI	HM	A	PE	EE	E	I	O
*N*	140	140	140	140	140	138 *	140	140
Mean	2.38	3.34	1.71	3.03	2.32	3.33	3.43	3.88
SD	0.81	0.95	0.80	0.84	0.86	0.76	0.89	0.77
Minimum	1.00	1.00	1.00	1.00	1.00	1.00	1.00	1.00
Maximum	5.00	5.00	4.00	5.00	5.00	5.00	5.00	5.00

Abbreviations: SI = social influence; HM = hedonic motivation; A = anthropomorphism; PE = performance expectancy; EE = effort expectancy; E = emotion; I = intent/willingness to accept the use of Artificial Intelligence devices; O = objection to the use of AI devices. * Missing data: 2 cases for emotion.

**Table 3:** Items removed during expert review and rationale for exclusion.

Item ID	Construct	Main Reason for Exclusion
SI2	Social Influence	Low relevance, conceptual redundancy
HM1	Hedonic Motivation	Low relevance, conceptual redundancy
HM5	Hedonic Motivation	Low relevance, consistently low expert ratings

**Table 4:** Standardized factor loadings (β) and significance levels for the eight-factor Confirmatory Factor Analysis (CFA) model.

Latent	Item	β	SE	*p*	R^2^
Social Influence (SI)	si1	0.729	—	—	0.531
	si3	0.920	0.112	<0.001	0.847
	si4	0.757	0.092	<0.001	0.573
	si5	0.752	0.107	<0.001	0.565
	si6	0.638	0.114	<0.001	0.407
Hedonic Motivation (HM)	hm2	0.904	—	—	0.818
	hm3	0.919	0.026	<0.001	0.845
	hm4	0.872	0.035	<0.001	0.760
Anthropomorphism (A)	a1	0.832	—	—	0.692
	a2	0.898	0.067	<0.001	0.806
	a3	0.911	0.046	<0.001	0.831
	a4	0.956	0.046	<0.001	0.913
Performance Expectancy (PE)	pe1	0.837	—	—	0.701
	pe2	0.708	0.072	<0.001	0.502
	pe3	0.858	0.088	<0.001	0.737
	pe4	0.840	0.075	<0.001	0.705
Effort Expectancy (EE)	ee1	0.810	—	—	0.656
	ee2	0.804	0.065	<0.001	0.647
	ee3	0.946	0.077	<0.001	0.895
Emotion (E)	e1	0.822	—	—	0.676
	e2	0.824	0.055	<0.001	0.679
	e3	0.861	0.061	<0.001	0.742
	e4	0.824	0.054	<0.001	0.678
	e5	0.831	0.058	<0.001	0.691
Intention/Willingness (I)	i1	0.846	—	—	0.716
	i2	0.925	0.042	<0.001	0.856
	i3	0.904	0.043	<0.001	0.817
Objection (O)	o1	0.745	—	—	0.556
	o2	0.938	0.144	<0.001	0.881
	o3	0.785	0.108	<0.001	0.616
	o4	0.762	0.105	<0.001	0.581

Abbreviations: SE = unstandardized standard error; R^2^ = item reliability (squared multiple correlation).

**Table 5:** Reliability of the Italian version of Artificially Intelligent Device Use Acceptance (AIDUA-IT) subscales.

Subscale	Cronbach’s α	McDonald’s ω
Social Influence (SI)	0.823	0.827
Hedonic Motivation (HM)	0.896	0.896
Anthropomorphism (A)	0.889	0.902
Performance Expectancy (PE)	0.847	0.848
Effort Expectancy (EE)	0.852	0.858
Emotion (E)	0.882	0.886
Intention/Willingness (I)	0.882	0.884
Objection (O)	0.837	0.839

**Table 6:** Convergent validity of AIDUA-IT constructs based on Average Variance Extracted (AVE) and square root of AVE.

Subscale	AVE	√AVE
Social Influence	0.580	0.762
Hedonic Motivation	0.806	0.898
Anthropomorphism	0.805	0.897
Performance Expectancy	0.659	0.811
Effort Expectancy	0.732	0.856
Emotion	0.692	0.832
Intention/Willingness	0.798	0.893
Objection	0.657	0.811

**Table 7:** Intraclass Correlation Coefficient (ICC) for each subscale.

Subscale	ICC (2,1)	95% Confidence Interval	*p*-Value
Social Influence (SI)	0.891	0.771–1.000	<0.001
Hedonic Motivation (HM)	0.848	0.704–1.000	<0.001
Anthropomorphism (A)	0.847	0.701–1.000	<0.001
Performance Expectancy (PE)	0.888	0.789–1.000	<0.001
Effort Expectancy (EE)	0.807	0.648–0.990	<0.001
Emotion (E)	0.901	0.822–1.000	<0.001
Intention/Willingness (I)	0.891	0.773–1.000	<0.001
Objection (O)	0.878	0.783–0.990	<0.001

## Data Availability

The data that support the findings of this study are available from the corresponding author upon reasonable request.

## Supplementary Materials

The following supporting information can be downloaded at: https://www.mdpi.com/article/10.3390/jcm15041578/s1, File S1. AIDUA-IT Translation and Content Validity Workbook (Word file with multiple tables including I-CVI and S-CVI/Ave calculations, expert panel ratings, item-level revision traceability and AIDUA-IT Final Italian Version). File S2. AIDUA-IT Psychometric Analysis Workbook (Word file with multiple tables including item-level descriptive statistics). File S3. AIDUA-IT Additional Statistical Outputs (Word file with multiple tables including raw AVE and composite reliability calculations, Fornell–Larcker matrices).

## Abbreviations

The following abbreviations are used in this manuscript:

AIDUA	Artificially Intelligent Device Use Acceptance
AIDUA-IT	Italian version of the AIDUA scale
AI-TAM	Artificial Intelligence Technology Acceptance Model
AVE	Average Variance Extracted
CFA	Confirmatory Factor Analysis
CFI	Comparative Fit Index
CI	Confidence Interval
COSMIN	Consensus-based Standards for the Selection of Health Measurement Instruments
CVI	Content Validity Index
DWLS	Diagonally Weighted Least Squares
EE	Effort Expectancy
E	Emotion
HM	Hedonic Motivation
ICC	Intraclass Correlation Coefficient
I	Intention/Willingness to Use
I-CVI	Item-level Content Validity Index
ISS	Istituto Superiore di Sanità
O	Objection to Use
PE	Performance Expectancy
PNRR	National Recovery and Resilience Plan (Italy)
RMSEA	Root Mean Square Error of Approximation
S-CVI/Ave	Scale-level Content Validity Index (Average Method)
SI	Social Influence
SRMR	Standardized Root Mean Square Residual
STROBE	Strengthening the Reporting of Observational Studies in Epidemiology
TLI	Tucker–Lewis Index
UTAUT	Unified Theory of Acceptance and Use of Technology
WLSMV	Weighted Least Squares Mean and Variance Adjusted
