# Supplementary material for: Preliminary Validation of the Italian Version of the Artificially Intelligent Device Use Acceptance (AIDUA-IT) Scale: Cross-Cultural Adaptation and Psychometric Evaluation
Source: J Clin Med. 2026 Feb 17;15(4):1578. doi: 10.3390/jcm15041578 (PMC12941690; doi:10.3390/jcm15041578)
Supplement: Supplementary file 1 [file jcm-15-01578-s001.zip › File S1.pdf]

Preliminary Validation of the Italian version of the Artificially Intelligent Device Use Acceptance (AIDUA-IT) scale: cross-cultural adaptation and psychometric evaluation

Cavasin Giulia, Ocagli Honoria, Gregori Dario

File S1. AIDUA-IT Translation and Content Validity Workbook

This file contains detailed documentation of the translation, cultural adaptation, and content validity assessment of the AIDUA-IT scale.

Table of Contents

Table S1. Expert ratings (1–4) across content validity criteria for all AIDUA items (Version 1)..... 3

Table S2. Expert panel ratings and actions taken during content validation of the AIDUA-IT items (Version 1) 7

Table S3. Revised Questionnaire (Version 2) 14

Table S4. Cognitive Debriefing: Participant Ratings and Item Paraphrasing 15

Table S5. Cognitive debriefing findings: item-level comments from pilot participants and resulting wording modifications 21

Final Italian Version of the Artificially Intelligent Device Use Acceptance Scale (AIDUA-IT) 23

Table S1. Expert ratings (1–4) across content validity criteria for all AIDUA items (Version 1)

**Item/Experts**	G.C.	C.F.	S.F.	F.M.	M.T.	M.C.	J.B.
1. Usare dispositivi basati su IA riflette uno status symbol nella mia cerchia sociale (ad es. amici, famiglia e colleghi). [Relevance]	4	4	3	2	3	3	3
1. Usare dispositivi basati su IA riflette uno status symbol nella mia cerchia sociale (ad es. amici, famiglia e colleghi). [Clarity]	3	3	3	3	3	2	4
1. Usare dispositivi basati su IA riflette uno status symbol nella mia cerchia sociale (ad es. amici, famiglia e colleghi). [Cultural Adequacy]	4	4	2	2	3	3	4
1. Usare dispositivi basati su IA riflette uno status symbol nella mia cerchia sociale (ad es. amici, famiglia e colleghi). [Linguistic Appropriateness]	4	3	3	2	3	2	3
2. Le persone che influenzano il mio comportamento vorrebbero che utilizzassi dispositivi basati su IA. [Relevance]	2	1	1	3	3	4	2
2. Le persone che influenzano il mio comportamento vorrebbero che utilizzassi dispositivi basati su IA. [Clarity]	3	1	3	4	4	4	4
2. Le persone che influenzano il mio comportamento vorrebbero che utilizzassi dispositivi basati su IA. [Cultural Adequacy]	4	1	2	3	3	4	3
2. Le persone che influenzano il mio comportamento vorrebbero che utilizzassi dispositivi basati su IA. [Linguistic Appropriateness]	4	1	3	3	4	4	3
3. Le persone nella mia rete sociale che usano dispositivi basati su IA hanno più prestigio di quelle che non li usano. [Relevance]	3	4	3	2	3	4	4
3. Le persone nella mia rete sociale che usano dispositivi basati su IA hanno più prestigio di quelle che non li usano. [Clarity]	2	4	3	4	3	4	4
3. Le persone nella mia rete sociale che usano dispositivi basati su IA hanno più prestigio di quelle che non li usano. [Cultural Adequacy]	2	4	3	3	3	4	4
3. Le persone nella mia rete sociale che usano dispositivi basati su IA hanno più prestigio di quelle che non li usano. [Linguistic Appropriateness]	2	4	3	3	3	4	4
4. Le persone di cui stimo l'opinione preferirebbero che io utilizzassi dispositivi basati su IA. [Relevance]	3	4	1	4	3	4	3
4. Le persone di cui stimo l'opinione preferirebbero che io utilizzassi dispositivi basati su IA. [Clarity]	4	4	3	4	4	4	3
4. Le persone di cui stimo l'opinione preferirebbero che io utilizzassi dispositivi basati su IA. [Cultural Adequacy]	3	4	3	3	3	4	4
4. Le persone di cui stimo l'opinione preferirebbero che io utilizzassi dispositivi basati su IA. [Linguistic Appropriateness]	4	4	3	3	3	4	3
5. Le persone per me importanti mi incoraggerebbero a utilizzare tali dispositivi. [Relevance]	3	4	3	4	4	3	1
5. Le persone per me importanti mi incoraggerebbero a utilizzare tali dispositivi. [Clarity]	3	4	3	4	4	4	3
5. Le persone per me importanti mi incoraggerebbero a utilizzare tali dispositivi. [Cultural Adequacy]	4	4	3	4	4	4	2
5. Le persone per me importanti mi incoraggerebbero a utilizzare tali dispositivi. [Linguistic Appropriateness]	4	4	2	4	4	4	3
6. Le persone nella mia rete sociale che usano dispositivi di intelligenza artificiale hanno un profilo elevato. [Relevance]	2	4	2	3	3	4	3
6. Le persone nella mia rete sociale che usano dispositivi di intelligenza artificiale hanno un profilo elevato. [Clarity]	2	4	3	3	3	4	4
6. Le persone nella mia rete sociale che usano dispositivi di intelligenza artificiale hanno un profilo elevato. [Cultural Adequacy]	2	4	3	2	3	4	3
6. Le persone nella mia rete sociale che usano dispositivi di intelligenza artificiale hanno un profilo elevato. [Linguistic Appropriateness]	3	4	3	2	3	4	3
7: Mi diverto a interagire con l'IA. [Relevance]	1	4	1	4	4	3	2
7: Mi diverto a interagire con l'IA. [Clarity]	4	4	1	4	4	4	3
7: Mi diverto a interagire con l'IA. [Cultural Adequacy]	4	4	2	3	4	4	3
7: Mi diverto a interagire con l'IA. [Linguistic Appropriateness]	4	4	3	3	4	2	2
8. Interagire con dispositivi basati su IA è divertente. [Relevance]	4	4	2	4	3	4	3
8. Interagire con dispositivi basati su IA è divertente. [Clarity]	4	4	3	4	2	4	3
8. Interagire con dispositivi basati su IA è divertente. [Cultural Adequacy]	4	4	3	3	2	4	2
8. Interagire con dispositivi basati su IA è divertente. [Linguistic Appropriateness]	4	4	2	3	2	4	2
9. Interagire con l'IA fa passare il tempo. [Relevance]	2	4	1	3	3	4	3
9. Interagire con l'IA fa passare il tempo. [Clarity]	3	4	2	2	2	4	2
9. Interagire con l'IA fa passare il tempo. [Cultural Adequacy]	3	4	3	3	3	4	2
9. Interagire con l'IA fa passare il tempo. [Linguistic Appropriateness]	4	4	3	3	3	3	2
10. L'interazione con l'IA è piacevole. [Relevance]	3	3	2	4	2	3	4

10. L'interazione con l'IA è piacevole. [Clarity]	4	4	1	4	3	3	4
10. L'interazione con l'IA è piacevole. [Cultural Adequacy]	3	4	2	3	2	3	3
10. L'interazione con l'IA è piacevole. [Linguistic Appropriateness]	4	4	2	3	3	3	3
11. Il processo stesso di interazione è gradevole. [Relevance]	2	4	3	2	2	3	4
11. Il processo stesso di interazione è gradevole. [Clarity]	2	4	1	2	2	2	4
11. Il processo stesso di interazione è gradevole. [Cultural Adequacy]	2	4	3	2	2	4	3
11. Il processo stesso di interazione è gradevole. [Linguistic Appropriateness]	3	4	3	2	2	4	4
12. I dispositivi basati su IA hanno una propria mente. [Relevance]	2	4	1	3	3	4	4
12. I dispositivi basati su IA hanno una propria mente. [Clarity]	3	4	3	4	2	4	3
12. I dispositivi basati su IA hanno una propria mente. [Cultural Adequacy]	3	4	2	3	2	4	3
12. I dispositivi basati su IA hanno una propria mente. [Linguistic Appropriateness]	4	4	2	3	2	4	2
13. I dispositivi basati su IA possiedono coscienza. [Relevance]	3	4	3	3	2	4	4
13. I dispositivi basati su IA possiedono coscienza. [Clarity]	4	4	1	4	2	4	3
13. I dispositivi basati su IA possiedono coscienza. [Cultural Adequacy]	2	4	3	3	2	4	3
13. I dispositivi basati su IA possiedono coscienza. [Linguistic Appropriateness]	3	4	3	3	2	4	2
14. I dispositivi basati su IA hanno libero arbitrio. [Relevance]	3	4	3	3	2	4	4
14. I dispositivi basati su IA hanno libero arbitrio. [Clarity]	2	4	3	4	2	4	3
14. I dispositivi basati su IA hanno libero arbitrio. [Cultural Adequacy]	4	4	3	3	2	4	3
14. I dispositivi basati su IA hanno libero arbitrio. [Linguistic Appropriateness]	3	4	3	3	2	4	3
15. I dispositivi basati su IA proveranno emozioni. [Relevance]	3	4	1	3	2	2	4
15. I dispositivi basati su IA proveranno emozioni. [Clarity]	2	4	1	4	2	4	4
15. I dispositivi basati su IA proveranno emozioni. [Cultural Adequacy]	4	4	3	3	2	4	3
15. I dispositivi basati su IA proveranno emozioni. [Linguistic Appropriateness]	4	4	3	3	2	4	2
16. I dispositivi basati su IA sono più accurati degli esseri umani. [Relevance]	4	4	2	4	2	4	4
16. I dispositivi basati su IA sono più accurati degli esseri umani. [Clarity]	3	4	2	4	1	4	3
16. I dispositivi basati su IA sono più accurati degli esseri umani. [Cultural Adequacy]	4	4	3	3	2	4	3
16. I dispositivi basati su IA sono più accurati degli esseri umani. [Linguistic Appropriateness]	4	4	3	3	2	4	2
17. I dispositivi basati su IA sono più accurati con meno errori umani. [Relevance]	3	4	3	4	3	4	4
17. I dispositivi basati su IA sono più accurati con meno errori umani. [Clarity]	2	4	3	2	3	3	3
17. I dispositivi basati su IA sono più accurati con meno errori umani. [Cultural Adequacy]	4	4	3	2	2	4	2
17. I dispositivi basati su IA sono più accurati con meno errori umani. [Linguistic Appropriateness]	4	4	3	2	2	4	1
18. I dispositivi basati su IA offrono un servizio più coerente rispetto agli esseri umani. [Relevance]	4	4	1	4	3	4	4
18. I dispositivi basati su IA offrono un servizio più coerente rispetto agli esseri umani. [Clarity]	3	4	1	2	3	3	4
18. I dispositivi basati su IA offrono un servizio più coerente rispetto agli esseri umani. [Cultural Adequacy]	3	4	2	2	3	4	4
18. I dispositivi basati su IA offrono un servizio più coerente rispetto agli esseri umani. [Linguistic Appropriateness]	4	4	3	2	3	4	3
19. Le informazioni fornite dai dispositivi basati su IA sono più coerenti. [Relevance]	4	4	2	4	2	4	4
19. Le informazioni fornite dai dispositivi basati su IA sono più coerenti. [Clarity]	4	1	2	3	2	4	4
19. Le informazioni fornite dai dispositivi basati su IA sono più coerenti. [Cultural Adequacy]	4	4	2	3	2	4	3
19. Le informazioni fornite dai dispositivi basati su IA sono più coerenti. [Linguistic Appropriateness]	4	3	3	3	2	4	2
20. Usare dispositivi basati su IA richiede troppo tempo. [Relevance]	4	4	3	4	2	4	3
20. Usare dispositivi basati su IA richiede troppo tempo. [Clarity]	4	4	3	4	3	4	3
20. Usare dispositivi basati su IA richiede troppo tempo. [Cultural Adequacy]	4	4	3	4	2	4	3

20. Usare dispositivi basati su IA richiede troppo tempo. [Linguistic Appropriateness]	4	4	3	4	3	4	2
21. Utilizzare dispositivi basati su intelligenza artificiale nei servizi è troppo complesso da comprendere e da gestire. [Relevance]	4	4	3	4	2	4	4
21. Utilizzare dispositivi basati su intelligenza artificiale nei servizi è troppo complesso da comprendere e da gestire. [Clarity]	3	4	1	3	2	4	4
21. Utilizzare dispositivi basati su intelligenza artificiale nei servizi è troppo complesso da comprendere e da gestire. [Cultural Adequacy]	4	4	3	3	2	4	2
21. Utilizzare dispositivi basati su intelligenza artificiale nei servizi è troppo complesso da comprendere e da gestire. [Linguistic Appropriateness]	4	4	2	3	2	4	2
22. Mi serve troppo tempo per imparare a interagire con dispositivi basati su IA. [Relevance]	4	4	3	4	3	4	4
22. Mi serve troppo tempo per imparare a interagire con dispositivi basati su IA. [Clarity]	4	4	3	4	2	4	3
22. Mi serve troppo tempo per imparare a interagire con dispositivi basati su IA. [Cultural Adequacy]	4	4	3	4	3	4	3
22. Mi serve troppo tempo per imparare a interagire con dispositivi basati su IA. [Linguistic Appropriateness]	4	4	3	4	3	4	3
23. Quando utilizzo l'IA mi sento: Annoiato – rilassato. [Relevance]	2	4	2	4	3	4	4
23. Quando utilizzo l'IA mi sento: Annoiato – rilassato. [Clarity]	4	4	3	4	2	4	4
23. Quando utilizzo l'IA mi sento: Annoiato – rilassato. [Cultural Adequacy]	2	4	2	4	3	4	4
23. Quando utilizzo l'IA mi sento: Annoiato – rilassato. [Linguistic Appropriateness]	3	4	3	4	3	4	2
24. Quando utilizzo l'IA mi sento: Malinconico - appagato [Relevance]	2	4	1	4	3	3	4
24. Quando utilizzo l'IA mi sento: Malinconico - appagato [Clarity]	4	4	1	4	2	4	4
24. Quando utilizzo l'IA mi sento: Malinconico - appagato [Cultural Adequacy]	2	4	2	4	3	4	4
24. Quando utilizzo l'IA mi sento: Malinconico - appagato [Linguistic Appropriateness]	2	4	2	4	3	4	2
25. Quando utilizzo l'IA mi sento: Avvilito - speranzoso. [Relevance]	2	4	3	4	3	3	4
25. Quando utilizzo l'IA mi sento: Avvilito - speranzoso. [Clarity]	4	4	2	4	2	4	4
25. Quando utilizzo l'IA mi sento: Avvilito - speranzoso. [Cultural Adequacy]	2	4	3	4	2	4	4
25. Quando utilizzo l'IA mi sento: Avvilito - speranzoso. [Linguistic Appropriateness]	2	4	3	4	3	3	4
26. Quando utilizzo l'IA mi sento: Insoddisfatto - soddisfatto. [Relevance]	4	4	3	4	3	4	4
26. Quando utilizzo l'IA mi sento: Insoddisfatto - soddisfatto. [Clarity]	4	4	3	4	2	4	4
26. Quando utilizzo l'IA mi sento: Insoddisfatto - soddisfatto. [Cultural Adequacy]	3	4	3	4	2	4	4
26. Quando utilizzo l'IA mi sento: Insoddisfatto - soddisfatto. [Linguistic Appropriateness]	3	4	3	4	3	4	4
27. Quando utilizzo l'IA mi sento: Infastidito - compiaciuto. [Relevance]	3	4	1	4	3	4	4
27. Quando utilizzo l'IA mi sento: Infastidito - compiaciuto. [Clarity]	4	4	1	4	2	4	4
27. Quando utilizzo l'IA mi sento: Infastidito - compiaciuto. [Cultural Adequacy]	3	4	3	4	2	4	4
27. Quando utilizzo l'IA mi sento: Infastidito - compiaciuto. [Linguistic Appropriateness]	4	4	3	4	3	4	4
28. Sono disposto a ricevere servizi da dispositivi basati su IA. [Relevance]	4	4	1	4	3	4	4
28. Sono disposto a ricevere servizi da dispositivi basati su IA. [Clarity]	4	4	1	3	3	4	4
28. Sono disposto a ricevere servizi da dispositivi basati su IA. [Cultural Adequacy]	4	4	3	3	3	4	4
28. Sono disposto a ricevere servizi da dispositivi basati su IA. [Linguistic Appropriateness]	4	4	2	3	3	4	4
29. Sarò felice di interagire con dispositivi basati su IA. [Relevance]	4	4	3	4	3	4	4
29. Sarò felice di interagire con dispositivi basati su IA. [Clarity]	4	4	3	4	2	4	3
29. Sarò felice di interagire con dispositivi basati su IA. [Cultural Adequacy]	4	4	3	4	3	4	4
29. Sarò felice di interagire con dispositivi basati su IA. [Linguistic Appropriateness]	4	4	3	4	3	4	4
30. È probabile che interagisca con dispositivi basati su IA. [Relevance]	4	4	3	4	3	4	4
30. È probabile che interagisca con dispositivi basati su IA. [Clarity]	4	4	3	4	2	4	3
30. È probabile che interagisca con dispositivi basati su IA. [Cultural Adequacy]	4	4	3	4	3	4	2
30. È probabile che interagisca con dispositivi basati su IA. [Linguistic Appropriateness]	4	4	3	4	3	4	2

31. Le informazioni vengono elaborate in modo meno umanizzato. [Relevance]	3	4	1	4	3	4	4
31. Le informazioni vengono elaborate in modo meno umanizzato. [Clarity]	3	4	1	3	1	4	4
31. Le informazioni vengono elaborate in modo meno umanizzato. [Cultural Adequacy]	3	4	3	3	2	4	4
31. Le informazioni vengono elaborate in modo meno umanizzato. [Linguistic Appropriateness]	3	4	2	3	2	4	2
32. Preferisco il contatto umano nelle interazioni con i servizi. [Relevance]	4	4	2	4	3	4	4
32. Preferisco il contatto umano nelle interazioni con i servizi. [Clarity]	4	4	1	4	2	4	4
32. Preferisco il contatto umano nelle interazioni con i servizi. [Cultural Adequacy]	4	4	2	3	3	4	4
32. Preferisco il contatto umano nelle interazioni con i servizi. [Linguistic Appropriateness]	4	4	2	3	3	4	3
33. Le persone hanno bisogno di scambi emotivi durante le interazioni con i servizi. [Relevance]	4	4	2	4	3	4	4
33. Le persone hanno bisogno di scambi emotivi durante le interazioni con i servizi. [Clarity]	4	4	1	3	2	4	4
33. Le persone hanno bisogno di scambi emotivi durante le interazioni con i servizi. [Cultural Adequacy]	4	4	2	3	2	4	4
33. Le persone hanno bisogno di scambi emotivi durante le interazioni con i servizi. [Linguistic Appropriateness]	4	4	2	3	3	4	4
34. L'interazione con dispositivi basati su IA manca di contatto sociale. [Relevance]	3	4	3	4	3	4	4
34. L'interazione con dispositivi basati su IA manca di contatto sociale. [Clarity]	4	4	3	3	2	4	3
34. L'interazione con dispositivi basati su IA manca di contatto sociale. [Cultural Adequacy]	3	4	3	3	2	4	4
34. L'interazione con dispositivi basati su IA manca di contatto sociale. [Linguistic Appropriateness]	3	4	3	2	3	4	2

Note. Ratings were provided on a 4-point Likert scale (1 = not relevant/clear/adequate, 2 = somewhat relevant/clear/adequate, 3 = quite relevant/clear/adequate, 4 = highly relevant/clear/adequate). Content validity criteria: relevance, clarity, cultural adequacy, and linguistic appropriateness. Abbreviations: G.C., C.F., S.F., F.M., M.T., M.C., J.B. = expert panel members.

Table S2. Expert panel ratings and actions taken during content validation of the AIDUA-IT items (Version 1)

Item/Experts	G.C.	C.F.	S.F.	F.M.	M.T	M.C	J.B.	I-CVI	Decision	Rationale	Rewording
1. Usare dispositivi basati su IA riflette uno status symbol nella mia cerchia sociale (ad es. amici, famiglia e colleghi). [Relevance]	1	1	1	0	1	1	1	0,86	Revised	Revised in cultural adequacy and linguistic appropriateness	Usare dispositivi basati sull'intelligenza artificiale riflette uno status all'interno della mia rete sociale (ad esempio amici, famiglia e colleghi)
1. Usare dispositivi basati su IA riflette uno status symbol nella mia cerchia sociale (ad es. amici, famiglia e colleghi). [Clarity]	1	1	1	1	1	0	1	0,86			
1. Usare dispositivi basati su IA riflette uno status symbol nella mia cerchia sociale (ad es. amici, famiglia e colleghi). [Cultural Adequacy]	1	1	0	0	1	1	1	0,71			
1. Usare dispositivi basati su IA riflette uno status symbol nella mia cerchia sociale (ad es. amici, famiglia e colleghi). [Linguistic Appropriateness]	1	1	1	0	1	0	1	0,71			
2. Le persone che influenzano il mio comportamento vorrebbero che utilizzassi dispositivi basati su IA. [Relevance]	0	0	0	1	1	1	0	0,43	Removed	Critical relevance concerns, conceptually redundant item	
2. Le persone che influenzano il mio comportamento vorrebbero che utilizzassi dispositivi basati su IA. [Clarity]	1	0	1	1	1	1	1	0,86			
2. Le persone che influenzano il mio comportamento vorrebbero che utilizzassi dispositivi basati su IA. [Cultural Adequacy]	1	0	0	1	1	1	1	0,71			
2. Le persone che influenzano il mio comportamento vorrebbero che utilizzassi dispositivi basati su IA. [Linguistic Appropriateness]	1	0	1	1	1	1	1	0,86			
3. Le persone nella mia rete sociale che usano dispositivi basati su IA hanno più prestigio di quelle che non li usano. [Relevance]	1	1	1	0	1	1	1	0,86	Accepted		
3. Le persone nella mia rete sociale che usano dispositivi basati su IA hanno più prestigio di quelle che non li usano. [Clarity]	0	1	1	1	1	1	1	0,86			
3. Le persone nella mia rete sociale che usano dispositivi basati su IA hanno più prestigio di quelle che non li usano. [Cultural Adequacy]	0	1	1	1	1	1	1	0,86			
3. Le persone nella mia rete sociale che usano dispositivi basati su IA hanno più prestigio di quelle che non li usano. [Linguistic Appropriateness]	0	1	1	1	1	1	1	0,86			
4. Le persone di cui stimo l'opinione preferirebbero che io utilizzassi dispositivi basati su IA. [Relevance]	1	1	0	1	1	1	1	0,86	Accepted		
4. Le persone di cui stimo l'opinione preferirebbero che io utilizzassi dispositivi basati su IA. [Clarity]	1	1	1	1	1	1	1	1,00			
4. Le persone di cui stimo l'opinione preferirebbero che io utilizzassi dispositivi basati su IA. [Cultural Adequacy]	1	1	1	1	1	1	1	1,00			
4. Le persone di cui stimo l'opinione preferirebbero che io utilizzassi dispositivi basati su IA. [Linguistic Appropriateness]	1	1	1	1	1	1	1	1,00			

5. Le persone per me importanti mi incoraggerebbero a utilizzare tali dispositivi. [Relevance]	1	1	1	1	1	1	0	0,86	Accepted		
5. Le persone per me importanti mi incoraggerebbero a utilizzare tali dispositivi. [Clarity]	1	1	1	1	1	1	1	1,00			
5. Le persone per me importanti mi incoraggerebbero a utilizzare tali dispositivi. [Cultural Adequacy]	1	1	1	1	1	1	0	0,86			
5. Le persone per me importanti mi incoraggerebbero a utilizzare tali dispositivi. [Linguistic Appropriateness]	1	1	0	1	1	1	1	0,86			
6. Le persone nella mia rete sociale che usano dispositivi di intelligenza artificiale hanno un profilo elevato. [Relevance]	0	1	0	1	1	1	1	0,71	Revised	Concept simplified	Nella mia rete sociale, coloro che utilizzano l'intelligenza artificiale sono persone di spicco
6. Le persone nella mia rete sociale che usano dispositivi di intelligenza artificiale hanno un profilo elevato. [Clarity]	0	1	1	1	1	1	1	0,86			
6. Le persone nella mia rete sociale che usano dispositivi di intelligenza artificiale hanno un profilo elevato. [Cultural Adequacy]	0	1	1	0	1	1	1	0,71			
6. Le persone nella mia rete sociale che usano dispositivi di intelligenza artificiale hanno un profilo elevato. [Linguistic Appropriateness]	1	1	1	0	1	1	1	0,86			
7: Mi diverto a interagire con l'IA. [Relevance]	0	1	0	1	1	1	0	0,57	Removed	Critical relevance concerns, conceptually redundant item	
7: Mi diverto a interagire con l'IA. [Clarity]	1	1	0	1	1	1	1	0,86			
7: Mi diverto a interagire con l'IA. [Cultural Adequacy]	1	1	0	1	1	1	1	0,86			
7: Mi diverto a interagire con l'IA. [Linguistic Appropriateness]	1	1	1	1	1	0	0	0,71			
8. Interagire con dispositivi basati su IA è divertente. [Relevance]	1	1	0	1	1	1	1	0,86	Revised	Concept simplified	Interagire con l'intelligenza artificiale è divertente
8. Interagire con dispositivi basati su IA è divertente. [Clarity]	1	1	1	1	0	1	1	0,86			
8. Interagire con dispositivi basati su IA è divertente. [Cultural Adequacy]	1	1	1	1	0	1	0	0,71			
8. Interagire con dispositivi basati su IA è divertente. [Linguistic Appropriateness]	1	1	0	1	0	1	0	0,57			
9. Interagire con l'IA fa passare il tempo. [Relevance]	0	1	0	1	1	1	1	0,71	Revised	Concept aligned more closely with original meaning	Interagire con l'intelligenza artificiale è stimolante
9. Interagire con l'IA fa passare il tempo. [Clarity]	1	1	0	0	0	1	0	0,43			
9. Interagire con l'IA fa passare il tempo. [Cultural Adequacy]	1	1	1	1	1	1	0	0,86			
9. Interagire con l'IA fa passare il tempo. [Linguistic Appropriateness]	1	1	1	1	1	1	0	0,86			
10. L'interazione con l'IA è piacevole. [Relevance]	1	1	0	1	0	1	1	0,71	Revised	Concept simplified	Interagire con l'intelligenza artificiale è piacevole
10. L'interazione con l'IA è piacevole. [Clarity]	1	1	0	1	1	1	1	0,86			
10. L'interazione con l'IA è piacevole. [Cultural Adequacy]	1	1	0	1	0	1	1	0,71			
10. L'interazione con l'IA è piacevole. [Linguistic Appropriateness]	1	1	0	1	1	1	1	0,86			
11. Il processo stesso di interazione è gradevole. [Relevance]	0	1	1	0	0	1	1	0,57	Removed	Consistently low ratings,	
11. Il processo stesso di interazione è gradevole. [Clarity]	0	1	0	0	0	0	1	0,29			
11. Il processo stesso di interazione è gradevole. [Cultural Adequacy]	0	1	1	0	0	1	1	0,57			

11. Il processo stesso di interazione è gradevole. [Linguistic Appropriateness]	1	1	1	0	0	1	1	0,71		conceptually redundant item	
12. I dispositivi basati su IA hanno una propria mente. [Relevance]	0	1	0	1	1	1	1	0,71	Revised	Wording made more direct	L'intelligenza artificiale possiede una propria mente
12. I dispositivi basati su IA hanno una propria mente. [Clarity]	1	1	1	1	0	1	1	0,86			
12. I dispositivi basati su IA hanno una propria mente. [Cultural Adequacy]	1	1	0	1	0	1	1	0,71			
12. I dispositivi basati su IA hanno una propria mente. [Linguistic Appropriateness]	1	1	0	1	0	1	0	0,57			
13. I dispositivi basati su IA possiedono coscienza. [Relevance]	1	1	1	1	0	1	1	0,86	Revised	Relevant concept, wording made more direct	L'intelligenza artificiale possiede una propria coscienza
13. I dispositivi basati su IA possiedono coscienza. [Clarity]	1	1	0	1	0	1	1	0,71			
13. I dispositivi basati su IA possiedono coscienza. [Cultural Adequacy]	0	1	1	1	0	1	1	0,71			
13. I dispositivi basati su IA possiedono coscienza. [Linguistic Appropriateness]	1	1	1	1	0	1	0	0,71			
14. I dispositivi basati su IA hanno libero arbitrio. [Relevance]	1	1	1	1	0	1	1	0,86	Accepted		
14. I dispositivi basati su IA hanno libero arbitrio. [Clarity]	0	1	1	1	0	1	1	0,71			
14. I dispositivi basati su IA hanno libero arbitrio. [Cultural Adequacy]	1	1	1	1	0	1	1	0,86			
14. I dispositivi basati su IA hanno libero arbitrio. [Linguistic Appropriateness]	1	1	1	1	0	1	1	0,86			
15. I dispositivi basati su IA proveranno emozioni. [Relevance]	1	1	0	1	0	1	1	0,71	Revised	Critical clarity concerns; concept shortened	L'intelligenza artificiale prova emozioni
15. I dispositivi basati su IA proveranno emozioni. [Clarity]	0	1	0	1	0	1	1	0,57			
15. I dispositivi basati su IA proveranno emozioni. [Cultural Adequacy]	1	1	1	1	0	1	1	0,86			
15. I dispositivi basati su IA proveranno emozioni. [Linguistic Appropriateness]	1	1	1	1	0	1	0	0,71			
16. I dispositivi basati su IA sono più accurati degli esseri umani. [Relevance]	1	1	0	1	0	1	1	0,71	Revised		Le risposte fornite dall'AI sono più accurate di quelle fornite dagli esseri umani
16. I dispositivi basati su IA sono più accurati degli esseri umani. [Clarity]	1	1	0	1	0	1	1	0,71			
16. I dispositivi basati su IA sono più accurati degli esseri umani. [Cultural Adequacy]	1	1	1	1	0	1	1	0,86			
16. I dispositivi basati su IA sono più accurati degli esseri umani. [Linguistic Appropriateness]	1	1	1	1	0	1	0	0,71			
17. I dispositivi basati su IA sono più accurati con meno errori umani. [Relevance]	1	1	1	1	1	1	1	1,00	Revised	Issues with linguistic appropriateness and cultural relevance, yet	I dispositivi basati sull'AI commettono meno errori rispetto agli umani
17. I dispositivi basati su IA sono più accurati con meno errori umani. [Clarity]	0	1	1	0	1	1	1	0,71			
17. I dispositivi basati su IA sono più accurati con meno errori umani. [Cultural Adequacy]	1	1	1	0	0	1	0	0,57			

17. I dispositivi basati su IA sono più accurati con meno errori umani. [Linguistic Appropriateness]	1	1	1	0	0	1	0	0,57		concept considered central	
18. I dispositivi basati su IA offrono un servizio più coerente rispetto agli esseri umani. [Relevance]	1	1	0	1	1	1	1	0,86	Revised	Limited clarity regarding the concept of "consistency"; item retained due to conceptual relevance	I dispositivi basati sull'AI offrono un servizio più costante rispetto agli essere umani
18. I dispositivi basati su IA offrono un servizio più coerente rispetto agli esseri umani. [Clarity]	1	1	0	0	1	1	1	0,71			
18. I dispositivi basati su IA offrono un servizio più coerente rispetto agli esseri umani. [Cultural Adequacy]	1	1	0	0	1	1	1	0,71			
18. I dispositivi basati su IA offrono un servizio più coerente rispetto agli esseri umani. [Linguistic Appropriateness]	1	1	1	0	1	1	1	0,86			
19. Le informazioni fornite dai dispositivi basati su IA sono più coerenti. [Relevance]	1	1	0	1	0	1	1	0,71	Revised	Clarity concerns due to the absence of an explicit comparison term	Le informazioni fornite dall'intelligenza artificiale sono più stabili e coerenti rispetto a quelle fornite dagli umani
19. Le informazioni fornite dai dispositivi basati su IA sono più coerenti. [Clarity]	1	0	0	1	0	1	1	0,57			
19. Le informazioni fornite dai dispositivi basati su IA sono più coerenti. [Cultural Adequacy]	1	1	0	1	0	1	1	0,71			
19. Le informazioni fornite dai dispositivi basati su IA sono più coerenti. [Linguistic Appropriateness]	1	1	1	1	0	1	0	0,71			
20. Usare dispositivi basati su IA mi richiede troppo tempo. [Relevance]	1	1	1	1	0	1	1	0,86	Accepted		
20. Usare dispositivi basati su IA mi richiede troppo tempo. [Clarity]	1	1	1	1	1	1	1	1,00			
20. Usare dispositivi basati su IA mi richiede troppo tempo. [Cultural Adequacy]	1	1	1	1	0	1	1	0,86			
20. Usare dispositivi basati su IA mi richiede troppo tempo. [Linguistic Appropriateness]	1	1	1	1	1	1	0	0,86			
21. Utilizzare dispositivi basati su intelligenza artificiale nei servizi è troppo complesso da comprendere e da gestire. [Relevance]	1	1	1	1	0	1	1	0,86	Revised	Relevant concept but unclear and linguistically problematic; wording simplified.	Nell'ambito dei servizi, utilizzare i dispoditivi basati su IA è troppo complesso
21. Utilizzare dispositivi basati su intelligenza artificiale nei servizi è troppo complesso da comprendere e da gestire. [Clarity]	1	1	0	1	0	1	1	0,71			
21. Utilizzare dispositivi basati su intelligenza artificiale nei servizi è troppo complesso da comprendere e da gestire. [Cultural Adequacy]	1	1	1	1	0	1	0	0,71			
21. Utilizzare dispositivi basati su intelligenza artificiale nei servizi è troppo complesso da comprendere e da gestire. [Linguistic Appropriateness]	1	1	0	1	0	1	0	0,57			
22. Mi serve troppo tempo per imparare a interagire con dispositivi basati su IA. [Relevance]	1	1	1	1	1	1	1	1,00	Accepted		
22. Mi serve troppo tempo per imparare a interagire con dispositivi basati su IA. [Clarity]	1	1	1	1	0	1	1	0,86			

22. Mi serve troppo tempo per imparare a interagire con dispositivi basati su IA. [Cultural Adequacy]	1	1	1	1	1	1	1	1,00			
22. Mi serve troppo tempo per imparare a interagire con dispositivi basati su IA. [Linguistic Appropriateness]	1	1	1	1	1	1	1	1,00			
23. Quando utilizzo l'IA mi sento: Annoiato – rilassato. [Relevance]	0	1	0	1	1	1	1	0,71	Revised	Limited cultural adequacy and relevance; adjectives revised for clarity and appropriateness.	Quando utilizzo l'intelligenza artificiale mi sento: nervoso - rilassato
23. Quando utilizzo l'IA mi sento: Annoiato – rilassato. [Clarity]	1	1	1	1	0	1	1	0,86			
23. Quando utilizzo l'IA mi sento: Annoiato – rilassato. [Cultural Adequacy]	0	1	0	1	1	1	1	0,71			
23. Quando utilizzo l'IA mi sento: Annoiato – rilassato. [Linguistic Appropriateness]	1	1	1	1	1	1	0	0,86			
24. Quando utilizzo l'IA mi sento: Malinconico - appagato [Relevance]	0	1	0	1	1	1	1	0,71	Revised	Linguistic appropriateness concerns and unclear meaning; adjectives simplified.	Quando utilizzo l'intelligenza artificiale mi sento: triste - contento
24. Quando utilizzo l'IA mi sento: Malinconico - appagato [Clarity]	1	1	0	1	0	1	1	0,71			
24. Quando utilizzo l'IA mi sento: Malinconico - appagato [Cultural Adequacy]	0	1	0	1	1	1	1	0,71			
24. Quando utilizzo l'IA mi sento: Malinconico - appagato [Linguistic Appropriateness]	0	1	0	1	1	1	0	0,57			
25. Quando utilizzo l'IA mi sento: Avvilito - speranzoso. [Relevance]	0	1	1	1	1	1	1	0,86	Revised	Concept unclear; adjectives streamlined to improve clarity.	Quando utilizzo l'intelligenza artificiale mi sento: sconfortato - confortato
25. Quando utilizzo l'IA mi sento: Avvilito - speranzoso. [Clarity]	1	1	0	1	0	1	1	0,71			
25. Quando utilizzo l'IA mi sento: Avvilito - speranzoso. [Cultural Adequacy]	0	1	1	1	0	1	1	0,71			
25. Quando utilizzo l'IA mi sento: Avvilito - speranzoso. [Linguistic Appropriateness]	0	1	1	1	1	1	1	0,86			
26. Quando utilizzo l'IA mi sento: Insoddisfatto - soddisfatto. [Relevance]	1	1	1	1	1	1	1	1,00	Accepted		
26. Quando utilizzo l'IA mi sento: Insoddisfatto - soddisfatto. [Clarity]	1	1	1	1	0	1	1	0,86			
26. Quando utilizzo l'IA mi sento: Insoddisfatto - soddisfatto. [Cultural Adequacy]	1	1	1	1	0	1	1	0,86			
26. Quando utilizzo l'IA mi sento: Insoddisfatto - soddisfatto. [Linguistic Appropriateness]	1	1	1	1	1	1	1	1,00			
27. Quando utilizzo l'IA mi sento: Infastidito - compiaciuto. [Relevance]	1	1	0	1	1	1	1	0,86	Accepted		
27. Quando utilizzo l'IA mi sento: Infastidito - compiaciuto. [Clarity]	1	1	0	1	0	1	1	0,71			
27. Quando utilizzo l'IA mi sento: Infastidito - compiaciuto. [Cultural Adequacy]	1	1	1	1	0	1	1	0,86			
27. Quando utilizzo l'IA mi sento: Infastidito - compiaciuto. [Linguistic Appropriateness]	1	1	1	1	1	1	1	1,00			
28. Sono disposto a ricevere servizi da dispositivi basati su IA. [Relevance]	1	1	0	1	1	1	1	0,86	Accepted		
28. Sono disposto a ricevere servizi da dispositivi basati su IA. [Clarity]	1	1	0	1	1	1	1	0,86			
28. Sono disposto a ricevere servizi da dispositivi basati su IA. [Cultural Adequacy]	1	1	1	1	1	1	1	1,00			

28. Sono disposto a ricevere servizi da dispositivi basati su IA. [Linguistic Appropriateness]	1	1	0	1	1	1	1	0,86			
29. Sarò felice di interagire con dispositivi basati su IA. [Relevance]	1	1	1	1	1	1	1	1,00	Accepted		
29. Sarò felice di interagire con dispositivi basati su IA. [Clarity]	1	1	1	1	0	1	1	0,86			
29. Sarò felice di interagire con dispositivi basati su IA. [Cultural Adequacy]	1	1	1	1	1	1	1	1,00			
29. Sarò felice di interagire con dispositivi basati su IA. [Linguistic Appropriateness]	1	1	1	1	1	1	1	1,00			
30. È probabile che interagisca con dispositivi basati su IA. [Relevance]	1	1	1	1	1	1	1	1,00	Accepted		
30. È probabile che interagisca con dispositivi basati su IA. [Clarity]	1	1	1	1	0	1	1	0,86			
30. È probabile che interagisca con dispositivi basati su IA. [Cultural Adequacy]	1	1	1	1	1	1	0	0,86			
30. È probabile che interagisca con dispositivi basati su IA. [Linguistic Appropriateness]	1	1	1	1	1	1	0	0,86			
31. Le informazioni vengono elaborate in modo meno umanizzato. [Relevance]	1	1	0	1	1	1	1	0,86	Revised	Linguistic appropriateness concerns, but the item was deemed conceptually relevant.	Le informazioni vengono elaborate dall'AI in modo poco umano
31. Le informazioni vengono elaborate in modo meno umanizzato. [Clarity]	1	1	0	1	0	1	1	0,71			
31. Le informazioni vengono elaborate in modo meno umanizzato. [Cultural Adequacy]	1	1	1	1	0	1	1	0,86			
31. Le informazioni vengono elaborate in modo meno umanizzato. [Linguistic Appropriateness]	1	1	0	1	0	1	0	0,57			
32. Preferisco il contatto umano nelle interazioni con i servizi. [Relevance]	1	1	0	1	1	1	1	0,86	Accepted		
32. Preferisco il contatto umano nelle interazioni con i servizi. [Clarity]	1	1	0	1	0	1	1	0,71			
32. Preferisco il contatto umano nelle interazioni con i servizi. [Cultural Adequacy]	1	1	0	1	1	1	1	0,86			
32. Preferisco il contatto umano nelle interazioni con i servizi. [Linguistic Appropriateness]	1	1	0	1	1	1	1	0,86			
33. Le persone hanno bisogno di scambi emotivi durante le interazioni con i servizi. [Relevance]	1	1	0	1	1	1	1	0,86	Revised	Conceptually relevant, but required specification of the service context and simplification of complex terminology.	Nell'ambito dei servizi, le persone hanno bisogno di scambi emotivi
33. Le persone hanno bisogno di scambi emotivi durante le interazioni con i servizi. [Clarity]	1	1	0	1	0	1	1	0,71			
33. Le persone hanno bisogno di scambi emotivi durante le interazioni con i servizi. [Cultural Adequacy]	1	1	0	1	0	1	1	0,71			
33. Le persone hanno bisogno di scambi emotivi durante le interazioni con i servizi. [Linguistic Appropriateness]	1	1	0	1	1	1	1	0,86			
34. L'interazione con dispositivi basati su IA manca di contatto sociale. [Relevance]	1	1	1	1	1	1	1	1,00	Accepted		
34. L'interazione con dispositivi basati su IA manca di contatto sociale. [Clarity]	1	1	1	1	0	1	1	0,86			

34. L'interazione con dispositivi basati su IA manca di contatto sociale. [Cultural Adequacy]	1	1	1	1	0	1	1	0,86			
34. L'interazione con dispositivi basati su IA manca di contatto sociale. [Linguistic Appropriateness]	1	1	1	0	1	1	0	0,71			
**S-CVI relevance**	**0,82**										
**S-CVI clarity**	**0,78**										
**S-CVI cultural adequacy**	**0,80**										
**S-CVI linguistic appropriateness**	**0,80**										

Note. For the purpose of content validity analysis, ratings were dichotomized as follows: scores of 1 or 2 were recoded as 0 (not adequate), and scores of 3 or 4 were recoded as 1 (adequate). I-CVI = Item Content Validity Index, calculated as the proportion of experts assigning a rating of 1 to the item. Decision rules: I-CVI ≥ 0.78 → item accepted; 0.70 ≤ I-CVI < 0.78 → item revised; I-CVI < 0.70 → item removed. At the end of the table S-CVI are presented.

Abbreviations: I-CVI = Item Content Validity Index.

Legend:

I-CVI 0.70-0.77
I-CVI <0.70
I-CVI ≥0.78

Table S3. Revised Questionnaire (Version 2)

1.	Usare dispositivi basati sull'intelligenza artificiale riflette uno status all'interno della mia rete sociale (ad esempio amici, famiglia e colleghi)
2.	/
3.	Le persone nella mia rete sociale che usano dispositivi basati su IA hanno più prestigio di quelle che non li usano.
4.	Le persone di cui stimo l'opinione preferirebbero che io utilizzassi dispositivi basati su IA.
5.	Le persone per me importanti mi incoraggerebbero a utilizzare tali dispositivi.
6.	Nella mia rete sociale, coloro che utilizzano l'intelligenza artificiale sono persone di spicco
7.	/
8.	Interagire con l'intelligenza artificiale è divertente
9.	Interagire con l'intelligenza artificiale è stimolante
10.	Interagire con l'intelligenza artificiale è piacevole
11.	/
12.	L'intelligenza artificiale possiede una propria mente
13.	L'intelligenza artificiale possiede una propria coscienza
14.	I dispositivi basati su IA hanno libero arbitrio.
15.	L'intelligenza artificiale prova emozioni
16.	Le risposte fornite dall'AI sono più accurate di quelle fornite dagli esseri umani
17.	I dispositivi basati sull'AI commettono meno errori rispetto agli umani
18.	I dispositivi basati sull'AI offrono un servizio più costante rispetto agli esseri umani
19.	Le informazioni fornite dall'intelligenza artificiale sono più stabili e coerenti rispetto a quelle fornite dagli umani
20.	Usare dispositivi basati su IA mi richiede troppo tempo.
21.	Nell'ambito dei servizi, utilizzare i dispositivi basati su IA è troppo complesso
22.	Mi serve troppo tempo per imparare a interagire con dispositivi basati su IA.
23.	Quando utilizzo l'intelligenza artificiale mi sento: nervoso - rilassato
24.	Quando utilizzo l'intelligenza artificiale mi sento: triste - contento
25.	Quando utilizzo l'intelligenza artificiale mi sento: sconfortato - confortato
26.	Quando utilizzo l'intelligenza artificiale mi sento: insoddisfatto - soddisfatto
27.	Quando utilizzo l'intelligenza artificiale mi sento: infastidito - compiaciuto
28.	Sono disposto a ricevere servizi da dispositivi basati su IA.
29.	Sarò felice di interagire con dispositivi basati su IA.
30.	È probabile che interagisca con dispositivi basati su IA.
31.	Le informazioni vengono elaborate dall'AI in modo poco umano
32.	Preferisco il contatto umano nelle interazioni con i servizi.
33.	Nell'ambito dei servizi, le persone hanno bisogno di scambi emotivi
34.	L'interazione con dispositivi basati su IA manca di contatto sociale.

Note. This table reports the revised Italian wording of all items retained after content validation and expert review (Version 2).
Legend:

Revised
Accepted

Table S4. Cognitive Debriefing: Participant Ratings and Item Paraphrasing

ID	Response_1	Response _2	Response _3	Response _4	Response _5	Response _6	Response _7	Response _8
**Age (years)**	52	70	58	22	31	73	62	49
**Gender**	Female	Male	Male	Female	Male	Female	Male	Female
**Educational level**	Bachelor's degree	High school diploma	High school diploma	High school diploma	Bachelor's degree	Primary school certificate	Middle school certificate	Bachelor's degree
**Occupation**	Administrative employee	Former teacher	Operations manager	Student	Station master	Former factory worker	Retired teacher	Nurse
**Computer skills (self-rated)**	Excellent	Fair	Excellent	Good	Good	Poor	Poor	Good
**Main purpose of device use**	Work	Leisure	Work	Leisure	Leisure	None	None	Leisure
**Weekly device use (hours)**	5-10	1-5	>15	5-10	1-5	Less than 1	1-5	1-5
Item 1 - Rating	3	1	3	4	1	1	2	2
Item 1 - Comment	Tra le persone che frequento coloro che utilizzano l'intelligenza artificiale appartengono ad una determinata categoria sociale	Usare dispositivi basati sull'intelligenza artificiale dimostra una certa posizione sociale all'interno della mia rete sociale.	Utilizzare l'AI mi aiuta a darmi lustro con i miei amici	Utilizzare l'AI mi rende più capace e intelligente rispetto agli altri	Penso che la domanda chieda se l'utilizzo della IA sia uno specchio del ruolo che ho all'interno della mia rete sociale, ad esempio l'utilizzo potrebbe riflettere il fatto di avere una laurea o titoli di studio maggiori	Usare dispositivi IA riflette uno status sociale	Usare l'intelligenza artificiale fa sembrare una persona più importante davanti ad amici e familiari.	Usare IA riflette uno status sociale
Item 3 - Rating	5	1	4	4	1	1	2	2
Item 3 - Comment	Tra le persone che conosco, coloro che utilizzano l'intelligenza artificiale catturano l'interesse degli interlocutori	Le persone nella mia rete sociale che usano dispositivi basati su ia hanno sono considerati più avanzati o moderni.	Chi usa l'intelligenza artificiale sulla mia rete di conoscenza risultano essere più competenti	Chi usa l'AI sono più capaci e competenti nel loro lavoro	Le persone nella mia rete sociale che usano IA sono più stimate rispetto a chi non utilizza	Nella mia rete, chi usa IA ha più prestigio	Chi conosco e usa questi strumenti è visto meglio rispetto a chi non li usa.	Tra quelli che conosco chi usa IA ha più prestigio
Item 4 - Rating	3	1	4	5	1	1	2	3
Item 4 - Comment	Le persone che stimo mi incoraggiano all'uso	Le persone di cui stimo l'opinione si aspettano che io usi dispositivi basati su ia.	Le persone di cui ho fiducia e stima mi consigliano di usare l'AI	Le persone a me vicine (come colleghi) mi consigliano di utilizzare l'AI	La domanda risulta chiara	Le persone che stimo vorrebbero che io li usassi	Le persone di cui rispetto il parere vorrebbero che anche io li usassi.	Le persone che stimo vorrebbero che io la usassi

	dell'intelligenza artificiale							
Item 5 - Rating	4	3	3	4	1	1	2	3
Item 5 - Comment	Ascolto i consigli di chi mi incoraggia all'uso della tecnologia	Mi sembra si voglia esplorare se le persone per me importanti mi spingerebbero verso l'uso di tali dispositivi.	Le persone che ritengo importanti mi consigliano di utilizzare l'AI	Le persone a me vicine mi consigliano di utilizzare l'AI (anche famiglia)	La domanda risulta chiara	Le persone per me importanti mi incoraggerebbero a usarli	Le persone per me importanti mi direbbero di provarli.	Le persone importanti mi incoraggerebbero
Item 6 - Rating	3	2	4	4	1	1	2	2
Item 6 - Comment	L'uso dell'AI rappresenta un plus tra quelli che conosco	L'idea è capire quanto sia vero che coloro che utilizzano l'intelligenza artificiale sono figure rilevanti o rispettate.	Le persone che conosco con maggiori capacità intellettuali e cognitive sono coloro che utilizzano l'AI	Tra le persone che conosco, risultano più di successo quelli che usano l'AI	Non capisco se per persone di spicco si intendano persone importanti per me (genitori, figure di riferimento ecc..) o di spicco a livello sociale (es. imprenditori, dirigenti ecc...)	Chi usa IA è una persona di spicco nella mia rete	Chi usa l'intelligenza artificiale nella mia cerchia è considerato importante.	Chi usa IA è di spicco
Item 8 - Rating	3	3	5	5	3	1	2	4
Item 8 - Comment	L'IA offre momenti di svago	Interagire con l'intelligenza artificiale intrattiene o incuriosisce.	è divertente usare l'AI	Mi piace porre domande all'AI, lo trovo divertente	La domanda risulta chiara	Usare IA è divertente	Usare l'intelligenza artificiale può essere divertente.	Interagire con IA è divertente
Item 9 - Rating	5	3	5	5	4	1	2	4
Item 9 - Comment	L'uso dell'AI può offrire spunti di riflessione e professionali	L'idea è capire quanto sia vero che interagire con l'intelligenza artificiale intellettualmente interessante.	Dialogare con l'AI può essere formativo e istruttivo	Ricevere risposte dall'AI stimola in me nuovi pensieri	stimolante è un termine troppo vago, stimola la curiosità? oppure si intende che stimola ulteriormente l'interesse verso l'IA stessa?	Usare IA è stimolante	È qualcosa che stimola la mente.	Interagire con IA è stimolante
Item 10 - Rating	4	5	3	5	2	1	2	4
Item 10 - Comment	L'uso dell'IA non annoia	Interagire con l'intelligenza artificiale è	Cambiando tipi di domande all'AI ci si può divertire	Mi piace passare del tempo parlando con l'AI	La domanda risulta chiara	Usare IA è piacevole	È un'esperienza piacevole.	Interagire con IA è piacevole

		gradevole da usare.						
Item 12 - Rating	1	2	5	2	1	3	3	2
Item 12 - Comment	L'IA ha autonomia di pensiero	L'intelligenza artificiale ragiona come se fosse autonoma.	L'AI è in grado di formulare pensieri complessi utilizzando algoritmi	L'AI è dotata di propria coscienza	non è molto chiaro cosa si intende per mente. intende un cervello fisico? o la capacità di compiere ragionamenti in maniera autonoma? o di avere pensieri propri	L'IA ha una mente propria (difficile comprensione)	L'intelligenza artificiale sembra avere una mente propria.	L'IA ha una mente propria
Item 13 - Rating	1	5	2	2	1	3	3	2
Item 13 - Comment	L'AI ha una sua morale ed etica	Se l'intelligenza artificiale sia consapevole di sé	L'IA ha una propria etica	L'AI possiede una propria coscienza (uguale)	La domanda risulta chiara	L'IA ha una coscienza	Sembra avere coscienza.	L'IA ha una coscienza
Item 14 - Rating	1	1	1	1	1	3	3	2
Item 14 - Comment	L'intelligenza artificiale ha autonomia di pensiero e compie scelte	Qui ci si chiede se i dispositivi basati su ia decidono in modo indipendente.	Si cerca di capire se i dispositivi basati su intelligenza artificiale sono dotati di una propria autonomia	i dispositivi basati su intelligenza artificiale sono in grado di prendere decisioni autonome	La domanda risulta chiara	I dispositivi IA hanno libero arbitrio	Pare che possa decidere da sola.	L'IA ha libero arbitrio
Item 15 - Rating	1	5	1	1	1	3	3	4
Item 15 - Comment	L'intelligenza artificiale prova emozioni	Qui si ragiona se davvero l'intelligenza artificiale reagisce emotivamente.	L'AI prova emozioni	L'AI può provare felicità, rabbia, tristezza	La domanda risulta chiara	L'IA prova emozioni	Può avere emozioni come le persone.	L'IA prova emozioni
Item 16 - Rating	5	2	4	3	3	2	3	3
Item 16 - Comment	L'AI dà risposte più precise e complete	Le risposte fornite dall'ai sono più precise di quelle fornite dagli esseri umani.	Le risposte fornite sono più argomentate e ricche di contenuto	L'AI dà risposte ricche di dettagli e veritiere rispetto agli esseri umani	La domanda risulta chiara	Le risposte dell'IA sono più accurate	Le risposte dell'intelligenza artificiale sono più precise di quelle umane.	Le risposte dell'IA sono più accurate dei umani
Item 17 - Rating	3	4	3	3	3	2	3	4
Item 17 - Comment	L'AI sbaglia meno dell'uomo	Se i dispositivi basati su ia commettono più	L'AI commette meno errori dell'uomo	L'AI commette meno sbagli	La domanda risulta chiara	L'IA fa meno errori	Fa meno errori rispetto alle persone.	L'IA commette meno errori

		affidabili rispetto agli umani.						
Item 18 - Rating	5	4	5	5	3	2	3	4
Item 18 - Comment	L'IA garantisce sempre lo stesso standard di efficienza, diversamente dall'essere umano	I dispositivi basati su ia offrono un servizio maggior coerenza nei risultati rispetto agli esseri umani.	I dispositivi basati su IA sono sempre disponibili h24	L'IA è sempre disponibile a rispondere alle domande	La domanda risulta chiara	L'IA è più costante	Lavora in modo più regolare e continuo.	L'IA offre un servizio più costante
Item 19 - Rating	5	5	4	5	3	2	3	2
Item 19 - Comment	L'IA è più razionale dell'essere umano	Se le informazioni fornite dall'intelligenza artificiale hanno maggior continuità nelle risposte rispetto a quelle umane.	Le risposte date dall'AI sono più pertinenti e centrate sull'obiettivo rispetto a quelle degli umani	Le info dell'IA non sono intaccate da emozioni e sono tratte sempre dalle medesime fonti	La domanda risulta chiara	Le sue informazioni sono più stabili e coerenti	Le informazioni che dà sono più chiare e stabili.	Le informazioni fornite dell'IA sono più stabili/coerenti
Item 20 - Rating	3	3	3	1	1	5	4	2
Item 20 - Comment	L'uso dell'AI comporta troppo dispendio di tempo	Se usare dispositivi basati su ia mi richiede richiede molto impegno.	La ricerca su AI è troppo onerosa in termini di tempo	Ci metto più tempo a cercare le cose su IA rispetto che da altre fonti	La domanda risulta chiara	Usare IA richiede troppo tempo	Usare questi strumenti mi porta via troppo tempo.	Usare IA richiede troppo tempo
Item 21 - Rating	3	5	3	2	1	5	4	2
Item 21 - Comment	Usare l'AI nell'ambito dei servizi all'utenza è troppo complesso	Come utilizzare i dispositivi basati su ia è difficile da gestire.	Utilizzare l'AI nei servizi risulta essere complicato	Utilizzare l'AI nei servizi è più difficile rispetto ai sistemi tradizionali	La domanda risulta chiara	Nei servizi, usare IA è troppo complesso	Nei servizi, usarli è troppo complicato.	È troppo complicato
Item 22 - Rating	2	4	3	2	2	5	4	4
Item 22 - Comment	Imparare ad usare l'AI comporta un enorme dispendio di tempo	Mi sembra si voglia esplorare se mi serve richiede molto impegno per capire come usare con dispositivi basati su ia.	Serve una buona pratica e addestramento per usare l'AI	Le AI non sono intuitivi	La domanda risulta chiara	Serve troppo tempo per imparare	Ci metto troppo a imparare come funzionano.	Serve troppo tempo per imparare
Item 23 - Rating	5	5	4	4	1	2	4	2
Item 23 - Comment	ansioso - tranquillo	mi mette ansia o mi tranquillizza.	L'utilizzo della AI mi permette di arrivare l'obiettivo velocemente	l'Ai mi dà immediatamente le risposte che cerco	La domanda risulta chiara	Mi sento poco rilassata	Mi sento più nervoso che rilassato.	Mi sento rilassata quando uso IA
Item 24 - Rating	5	3	4	4	1	3	3	4

Item 24 - Comment	amareggiato - sereno	mi deprime o mi rallegra.	La ricerca e la risposta utilizzando l'AI mi permette di avere risultato e mi dà senso di soddisfazione	L'AI mi dà subito la risposta che cerco ed è quella sperata	La domanda risulta chiara	Non mi sento né triste né contenta	Più triste che contento.	Mi sento contenta
Item 25 - Rating	5	1	3	4	1	2	3	4
Item 25 - Comment	sconfitto - soddisfatto	mi lascia perplesso o rassicurato.	Quando conferma il mio pensiero	L'AI mi dà conferma di quello che pensavo	La domanda risulta chiara	Mi sento poco confortata	Più a disagio che tranquillo.	Mi sento confortata
Item 26 - Rating	4	1	4	4	4	2	3	4
Item 26 - Comment	annoiato - entusiasta (rispetto alle risposte)	non mi appaga o mi gratifica.	Quando ottengo argomentazioni sensati e con riferimenti precisi	Quando l'AI mi fornisce informazioni accurate con fonte citata su cui posso fidarmi	La domanda risulta chiara	Mi sento poco soddisfatta	Più insoddisfatto che soddisfatto.	Mi sento soddisfatta
Item 27 - Rating	4	3	4	3	1	2	4	4
Item 27 - Comment	infastidito - ammirato	mi disturba o mi piace.	Infastidito quando devo ripetere più volte l'argomento	Quando non capisce la domanda e devo ripeterla, compiaciuto quando mi dà ragione	La domanda risulta chiara	Mi sento poco compiaciuta	Più infastidito che felice.	Mi sento compiaciuta
Item 28 - Rating	5	4	4	5	4	2	3	4
Item 28 - Comment	Sono disponibile ad essere un utente servito da AI	Come sono disposto a usufruire di soluzioni offerte da dispositivi basati su ia.	Sono disposto ad utilizzare servizi con AI purchè non a pagamento	Uso l'AI per scopi di servizio	La domanda risulta chiara	Sono disposta a ricevere servizi da IA	Accetterei di ricevere servizi da questi strumenti.	Sono disposta a ricevere servizi da IA
Item 29 - Rating	5	3	4	5	4	1	2	4
Item 29 - Comment	Sono disponibile in futuro ad ottenere risposte dall'AI	Penso si stia parlando del fatto che sarò contento di usare dispositivi basati su ia.	Ritengo interessante e istruttivo interagire con l'Ai	Speranza che nel futuro venga usata su più domande	La domanda risulta chiara	Sarò felice di interagire con IA	Usarli potrebbe anche rendermi felice.	Sarò felice di interagire
Item 30 - Rating	5	4	3	3	4	1	2	4
Item 30 - Comment	Già adesso anche inconsapevolmente probabilmente	Qui si ragiona su se davvero potrei utilizzare spesso	c'è una buona probabilità che	è probabile che anche senza sapere sto già utilizzando	metterei all'inizio della domanda un	È probabile che io interagisca con IA	Forse potrei iniziare a usarli.	È probabile che interagisca

	utilizzo dispositivi AI.	con dispositivi basati su ia.	continui ad utilizzare l'AI	l'intelligenza artificiale	"se ne avessi la possibilità"			
Item 31 - Rating	3	4	4	1	4	4	4	3
Item 31 - Comment	L'IA interagisce con le persone in modo non umanizzato	Le informazioni elaborate dall'ai sono senza sensibilità.	Quanto riportato dall'AI è privo di emozione	Le risposte dell'intelligenza artificiale non contengono parole come: grazie, prego… (ambigua)	La domanda risulta chiara	Le informazioni dell'IA sono poco umane (vaga)	L'intelligenza artificiale non ragiona come una persona.	Le informazioni dell'IA sono poco umane
Item 32 - Rating	1	5	2	1	3	5	5	4
Item 32 - Comment	Nel richiedere servizi, preferisco interloquire con le persone piuttosto che AI	L'idea è capire quanto sia vero che preferisco l'interazione diretta con persone nelle interazioni con i servizi.	Preferisco il contatto visivo e con le persone al quale richiedo un servizio	Preferisco quando prenoto un servizio telefonare e ricevere risposta da una persona	La domanda risulta chiara	Preferisco il contatto umano	Preferisco parlare con una persona vera.	Preferisco il contatto umano
Item 33 - Rating	2	2	1	5	2	5	5	5
Item 33 - Comment	Nel richiedere servizi le persone hanno necessità di rapporto umano	Penso si stia parlando del fatto che nell'ambito dei servizi, le persone hanno bisogno di connessioni affettive.	Le persone che richiedono servizi hanno bisogno di emozioni	Quando le persone vanno ad appuntamenti hanno bisogno anche di conforto e di contatto umano	La domanda risulta chiara	Le persone hanno bisogno di scambi emotivi	Nei servizi, le persone hanno bisogno di emozioni e umanità.	Le persone hanno bisogno di scambi emotivi
Item 34 - Rating	2	5	3	5	5	5	5	4
Item 34 - Comment	Nell'interloquire con l'AI manca il contesto sociale	L'interazione con dispositivi basati su ia non coinvolge relazioni personali.	L'utilizzo di AI toglie la parte di contatto umano isolandoti all'interno del sistema informatico	Usare l'AI al posto di metodiche tradizionali fa sì che ci sia meno socialità	La domanda risulta chiara	L'interazione con IA manca di contatto sociale	Con questi strumenti manca il contatto umano.	L'interazione con IA manca di contatto sociale

Note. Participants from a heterogeneous sample completed the AIDUA-IT items and provided paraphrased interpretations of each statement to support the evaluation of item comprehension and conceptual equivalence. Yellow-shaded cells indicate items flagged as problematic.

Abbreviations: n = number of participants.

Table S5. Cognitive debriefing findings: item-level comments from pilot participants and resulting wording modifications

Item	Feedback from cognitive debriefing	Original wording (Italian)	Issues identified	Proposed revision (Italian)
**Item 1**	No relevant issues identified.			
**Item 3**	No relevant issues identified.			
**Item 4**	No relevant issues identified.			
**Item 5**	No relevant issues identified.			
**Item 6**	The term “persone di spicco” (prominent people) was perceived as ambiguous (social, family, or professional status?).	Nella mia rete sociale, coloro che utilizzano l'intelligenza artificiale sono persone di spicco	“Persone di spicco” is too vague and may be interpreted differently across contexts.	Nella mia rete sociale, chi utilizza dispositivi basati su intelligenza artificiale è visto come un professionista di alto profilo
**Item 8**	No relevant issues identified.			
**Item 9**	The term “stimolante” (stimulating) was perceived as generic and multidimensional.	Interagire con l’intelligenza artificiale è stimolante	“Stimolante” is too general: unclear what type of stimulation or engagement is implied.	Interagire con l’intelligenza artificiale è coinvolgente
**Item 10**	No relevant issues identified.			
**Item 12**	Conceptual ambiguity: “avere una propria mente” (having its own mind) could be interpreted metaphorically or literally.	L’intelligenza artificiale possiede una propria mente	Potential confusion between literal and metaphorical interpretation.	I dispositivi basati sull'intelligenza artificiale hanno una propria volontà
**Item 13**	No relevant issues identified.			
**Item 14**	No relevant issues identified.			
**Item 15**	No relevant issues identified.			
**Item 16**	No relevant issues identified.			
**Item 17**	No relevant issues identified.			
**Item 18**	No relevant issues identified.			
**Item 19**	No relevant issues identified.			
**Item 20**	No relevant issues identified.			
**Item 21**	No relevant issues identified.			
**Item 22**	No relevant issues identified.			
**Item 23**	No relevant issues identified.			
**Item 24**	No relevant issues identified.			
**Item 25**	No relevant issues identified.			
**Item 26**	No relevant issues identified.			
**Item 27**	No relevant issues identified.			
**Item 28**	No relevant issues identified.			

**Item 29**	No relevant issues identified.			
**Item 30**	Suggested to adopt a hypothetical form (conditional clause).	È probabile che interagisca con dispositivi basati su IA	The statement may sound too affirmative; suggestion for conditional phrasing.	Se ne ho la possibilità, è probabile che interagisca con dispositivi basati su IA
**Item 31**	The expression “modo poco umano” was perceived as unclear; lack of reference to tone, empathy, or language style.	Le informazioni vengono elaborate dall’AI in modo poco umano	Wording too vague and open to interpretation.	Le informazioni elaborate dell’intelligenza artificiale mi sembrano fredde o impersonali
**Item 32**	No relevant issues identified.			
**Item 33**	No relevant issues identified.			
**Item 34**	No relevant issues identified.			

Note. This table summarizes qualitative feedback from pilot participants and the corresponding wording modifications implemented by the research team.

Legend:

Item flagged as problematic
Item revised according to comments

Final Italian Version of the Artificially Intelligent Device Use Acceptance Scale (AIDUA-IT)

1. Usare dispositivi basati sull'intelligenza artificiale riflette uno status all'interno della mia rete sociale (ad esempio amici, famiglia e colleghi)
2. Le persone nella mia rete sociale che usano dispositivi basati su IA hanno più prestigio di quelle che non li usano.
3. Le persone di cui stimo l'opinione preferirebbero che io utilizzassi dispositivi basati su IA
4. Le persone per me importanti mi incoraggerebbero a utilizzare tali dispositivi
5. Nella mia rete sociale, chi utilizza dispositivi basati su intelligenza artificiale è visto come un professionista di alto profilo
6. Interagire con l'intelligenza artificiale è divertente
7. Interagire con l'intelligenza artificiale è coinvolgente
8. Interagire con l'intelligenza artificiale è piacevole
9. I dispositivi basati sull'intelligenza artificiale hanno una propria volontà
10. I dispositivi basati su IA possiedono una propria coscienza
11. I dispositivi basati su IA hanno libero arbitrio
12. I dispositivi basati su IA provano emozioni
13. Le risposte fornite dall'AI sono più accurate di quelle fornite dagli esseri umani
14. I dispositivi basati sull'AI commettono meno errori rispetto agli umani
15. I dispositivi basati sull'AI offrono un servizio più costante rispetto agli esseri umani
16. Le informazioni fornite dall'intelligenza artificiale sono più stabili e coerenti rispetto a quelle fornite dagli umani
17. Usare dispositivi basati su IA mi richiede troppo tempo
18. Nell'ambito dei servizi, utilizzare i dispositivi basati su IA è troppo complesso
19. Mi serve troppo tempo per imparare a interagire con dispositivi basati su IA
20. Quando utilizzo l'intelligenza artificiale mi sento: nervoso - rilassato
21. Quando utilizzo l'intelligenza artificiale mi sento: triste - contento
22. Quando utilizzo l'intelligenza artificiale mi sento: sconfortato - confortato
23. Quando utilizzo l'intelligenza artificiale mi sento: insoddisfatto - soddisfatto
24. Quando utilizzo l'intelligenza artificiale mi sento: infastidito - compiaciuto
25. Sono disposto a ricevere servizi da dispositivi basati su IA
26. Sarò felice di interagire con dispositivi basati su IA
27. Se ne ho la possibilità, è probabile che interagisca con dispositivi basati su IA
28. Le informazioni elaborate dell'intelligenza artificiale mi sembrano fredde o impersonali
29. Preferisco il contatto umano nelle interazioni con i servizi
30. Nell'ambito dei servizi, le persone hanno bisogno di scambi emotivi
31. L'interazione con dispositivi basati su IA manca di contatto sociale

Note. All items are rated on a 5-point Likert scale ranging from 1 = strongly disagree to 5 = strongly agree.
