# Supplementary material for: Preliminary Validation of the Italian Version of the Artificially Intelligent Device Use Acceptance (AIDUA-IT) Scale: Cross-Cultural Adaptation and Psychometric Evaluation
Source: J Clin Med. 2026 Feb 17;15(4):1578. doi: 10.3390/jcm15041578 (PMC12941690; doi:10.3390/jcm15041578)
Supplement: Supplementary file 1 [file jcm-15-01578-s001.zip › File S2.pdf]

Preliminary Validation of the Italian version of the Artificially Intelligent Device Use Acceptance (AIDUA-IT) scale: cross-cultural adaptation and psychometric evaluation

Cavasin Giulia, Ocagli Honoria, Gregori Dario

File S2. AIDUA-IT Psychometric Analysis Workbook

This supplementary file provides detailed documentation of the psychometric evaluation of the Italian version of the Artificially Intelligent Device Use Acceptance (AIDUA-IT) scale, including item-level descriptive statistics, correlation analyses, and full confirmatory factor analysis outputs.

Table of Contents

Table S6. Descriptive statistics (Mean, Median, SD, IQR) for each item of the AIDUA-IT (5-point Likert scale)........ 3

Table S7. Correlation matrix based on mean scores of items belonging to each AIDUA-IT latent construct (Pearson's r, 95% CI, and p-values). 4

Table S6. Descriptive statistics (Mean, Median, SD, IQR) for each item of the AIDUA-IT (5-point Likert scale).

Descriptives	N	Missing	Mean	Median	SD	IQR
si1	140	0	2.61	2.00	1.11	2.00
si3	140	0	2.17	2.00	1.08	2.00
si4	140	0	2.11	2.00	0.99	2.00
si5	140	0	2.64	2.00	1.05	2.00
si6	140	0	2.39	2.00	1.02	1.00
hm1	140	0	3.46	4.00	1.03	1.00
hm3	140	0	3.26	3.00	1.08	1.00
hm4	140	0	3.31	3.00	1.03	1.00
a1	140	0	2.05	2.00	1.09	2.00
a2	140	0	1.67	1.00	0.94	1.00
a3	140	0	1.73	1.00	0.94	1.00
a4	140	0	1.40	1.00	0.69	1.00
pe1	140	0	2.81	3.00	1.02	1.00
pe2	140	0	2.81	3.00	0.96	1.00
pe3	140	0	3.50	4.00	1.04	1.00
pe4	140	0	2.99	3.00	1.05	2.00
ee1	140	0	2.34	2.00	1.06	1.00
ee2	140	0	2.39	2.00	0.90	1.00
ee3	140	0	2.24	2.00	0.97	1.00
e1	139	1	3.45	3.00	1.03	1.00
e2	139	1	3.31	3.00	0.79	1.00
e3	139	1	3.24	3.00	0.82	1.00
e4	139	1	3.51	4.00	0.92	1.00
e5	138	2	3.12	3.00	0.99	1.00
i1	140	0	3.51	4.00	0.91	1.00
i2	140	0	3.24	3.00	1.02	1.00
i3	140	0	3.54	4.00	1.03	1.00
o1	140	0	3.22	3.00	1.00	2.00
o2	140	0	4.12	4.00	0.93	1.00
o3	140	0	4.06	4.00	0.98	1.00
o4	140	0	4.11	4.00	0.95	1.00

Note. All items were rated on a 5-point Likert scale ranging from 1 = strongly disagree to 5 = strongly agree.
Abbreviations.
SI = Social Influence
HM = Hedonic Motivation
A = Anthropomorphism
PE = Performance Expectancy
EE = Effort Expectancy
E = Emotion
I = Intention / Willingness to Use
O = Objection to Use
SD = Standard Deviation
IQR = Interquartile Range

Table S7. Correlation matrix based on mean scores of items belonging to each AIDUA-IT latent construct (Pearson's r, 95% CI, and p-values).

Correlation Matrix

		Mean_I	Mean_SI	Mean_HM	Mean_A	Mean_PE	Mean_EE	Mean_E	Mean_O
Mean_I	Pearson's r	—							
	df	—							
	p-value	—							
	95% CI Upper	—							
	95% CI Lower	—							
Mean_SI	Pearson's r	0.467***	—						
	df	138	—						
	p-value	<.001	—						
	95% CI Upper	0.587	—						
	95% CI Lower	0.326	—						
Mean_HM	Pearson's r	0.641***	0.338***	—					
	df	138	138	—					
	p-value	<.001	<.001	—					
	95% CI Upper	0.730	0.477	—					
	95% CI Lower	0.532	0.182	—					
Mean_A	Pearson's r	0.054	0.193*	0.190*	—				
	df	138	138	138	—				
	p-value	0.529	0.023	0.025	—				
	95% CI Upper	0.218	0.347	0.345	—				
	95% CI Lower	-0.113	0.028	0.025	—				
Mean_PE	Pearson's r	0.456***	0.367***	0.436***	0.057	—			
	df	138	138	138	138	—			
	p-value	<.001	<.001	<.001	0.501	—			
	95% CI Upper	0.578	0.502	0.561	0.221	—			
	95% CI Lower	0.313	0.214	0.291	-0.110	—			
Mean_EE	Pearson's r	-0.421***	0.021	-0.455***	0.294***	-0.107	—		
	df	138	138	138	138	138	—		

	p-value	<.001	0.809	<.001	<.001	0.210	—		
	95% CI Upper	-0.274	0.186	-0.312	0.438	0.060	—		
	95% CI Lower	-0.549	-0.146	-0.577	0.135	-0.268	—		
Mean_E	Pearson's r	0.525***	0.285***	0.707***	0.131	0.374***	-0.580***	—	
	df	136	136	136	136	136	136	—	
	p-value	<.001	<.001	<.001	0.125	<.001	<.001	—	
	95% CI Upper	0.637	0.432	0.781	0.292	0.509	-0.458	—	
	95% CI Lower	0.393	0.124	0.612	-0.037	0.221	-0.681	—	
Mean_O	Pearson's r	-0.538***	-0.332***	-0.392***	-0.049	-0.402***	0.348***	-0.450***	—
	df	138	138	138	138	138	138	136	—
	p-value	<.001	<.001	<.001	0.569	<.001	<.001	<.001	—
	95% CI Upper	-0.408	-0.176	-0.242	0.118	-0.253	0.486	-0.306	—
	95% CI Lower	-0.646	-0.472	-0.524	-0.213	-0.532	0.193	-0.574	—

Note. Values represent Pearson's correlation coefficients (r) based on mean scores of items for each latent construct. Two-tailed p-values and 95% confidence intervals (CI) are reported. Degrees of freedom (df) vary due to minimal missing data in the Emotion items.

Significance levels: * $p < .05$, ** $p < .01$, *** $p < .001$.

Abbreviations.

SI = Social Influence
HM = Hedonic Motivation
A = Anthropomorphism
PE = Performance Expectancy
EE = Effort Expectancy
E = Emotion
I = Intention / Willingness to Use
O = Objection to Use
CI = Confidence Interval
df = degrees of freedom
