# Supplementary material for: Preliminary Validation of the Italian Version of the Artificially Intelligent Device Use Acceptance (AIDUA-IT) Scale: Cross-Cultural Adaptation and Psychometric Evaluation
Source: J Clin Med. 2026 Feb 17;15(4):1578. doi: 10.3390/jcm15041578 (PMC12941690; doi:10.3390/jcm15041578)
Supplement: Supplementary file 1 [file jcm-15-01578-s001.zip › File S3.pdf]

# Preliminary Validation of the Italian version of the Artificially Intelligent Device Use Acceptance (AIDUA-IT) scale: cross-cultural adaptation and psychometric evaluation

Cavasin Giulia, Ocagli Honoria, Gregori Dario

# File S3. AIDUA-IT Additional Statistical Outputs

This supplementary file contains additional statistical outputs supporting the construct validity assessment of the AIDUA-IT scale, including Average Variance Extracted and composite reliability calculations, Fornell–Larcker discriminant validity matrices, and summary tables for convergent and discriminant validity.

## Table of Contents

Table S8. Item reliability ($R^2$), Average Variance Extracted (AVE), and square root of AVE for the AIDUA-IT latent constructs. .......... 3

Table S9. Discriminant validity matrix based on the Fornell–Larcker criterion for the AIDUA-IT latent constructs. .......... 4

Table S10. Discriminant validity assessment using the Fornell–Larcker criterion for AIDUA-IT subscales. .......... 5

**Table S8.** Item reliability ($R^2$), Average Variance Extracted (AVE), and square root of AVE for the AIDUA-IT latent constructs.

| Variable | $R^2$ | AVE | √AVE |
|---|---|---|---|
| si1 | 0.531 | 0.580 | 0.762 |
| si2 | 0.847 | | |
| si3 | 0.573 | | |
| si4 | 0.565 | | |
| si5 | 0.407 | | |
| hm1 | 0.818 | 0.806 | 0.898 |
| hm2 | 0.845 | | |
| hm3 | 0.760 | | |
| a1 | 0.692 | 0.805 | 0.897 |
| a2 | 0.806 | | |
| a3 | 0.831 | | |
| a4 | 0.913 | | |
| pe1 | 0.701 | 0.659 | 0.811 |
| pe2 | 0.502 | | |
| pe3 | 0.737 | | |
| pe4 | 0.705 | | |
| ee1 | 0.656 | 0.732 | 0.856 |
| ee2 | 0.647 | | |
| ee3 | 0.895 | | |
| e1 | 0.676 | 0.692 | 0.832 |
| e2 | 0.679 | | |
| e3 | 0.742 | | |
| e4 | 0.678 | | |
| e5 | 0.691 | | |
| i1 | 0.716 | 0.798 | 0.893 |
| i2 | 0.856 | | |
| i3 | 0.817 | | |
| o1 | 0.556 | 0.657 | 0.811 |
| o2 | 0.881 | | |
| o3 | 0.616 | | |
| o4 | 0.581 | | |

Note. $R^2$ represents the proportion of variance explained in each observed item by its corresponding latent construct. AVE represents the average variance extracted at the construct level. √AVE indicates the square root of AVE. Convergent validity thresholds: AVE ≥ 0.50 = good; 0.40–0.49 = marginal; < 0.40 = weak.

Abbreviations:
SI = Social Influence; HM = Hedonic Motivation; A = Anthropomorphism; PE = Performance Expectancy; EE = Effort Expectancy; E = Emotion; I = Intention / Willingness to Use; O = Objection to Use.

**Table S9.** Discriminant validity matrix based on the Fornell–Larcker criterion for the AIDUA-IT latent constructs.

| | SI | HM | A | PE | EE | E | I | O |
|---|---|---|---|---|---|---|---|---|
| SI Social Influence | **0.762** | | | | | | | |
| HM Hedonic Motivation | 0.338 | **0.898** | | | | | | |
| A Anthropomorphism | 0.193 | 0.190 | **0.897** | | | | | |
| PE Performance Expectancy | 0.367 | 0.436 | 0.057 | **0.811** | | | | |
| EE Effort Expectancy | 0.021 | -0.455 | 0.294 | -0.107 | **0.856** | | | |
| E Emotion | 0.285 | 0.707 | 0.131 | 0.374 | -0.580 | **0.832** | | |
| I Intention of use | 0.467 | 0.641 | 0.054 | 0.456 | -0.421 | 0.525 | **0.893** | |
| O Objection | -0.332 | -0.392 | -0.049 | -0.402 | 0.348 | -0.450 | -0.538 | **0.811** |

Note. Diagonal values represent the square root of the Average Variance Extracted (√AVE) for each construct. Off-diagonal values represent latent construct correlations. Discriminant validity is supported when the square root of AVE for each construct exceeds its correlations with all other constructs, according to the Fornell–Larcker criterion.

Abbreviations:
SI = Social Influence;
HM = Hedonic Motivation;
A = Anthropomorphism;
PE = Performance Expectancy;
EE = Effort Expectancy;
E = Emotion;
I = Intention / Willingness to Use;
O = Objection to Use.

**Table S10.** Discriminant validity assessment using the Fornell–Larcker criterion for AIDUA-IT subscales.

| **Subscale** | **AVE** | **√AVE** | **Highest inter-factor correlation (r)** | **√AVE > r?** | **Interpretation*** |
|---|---|---|---|---|---|
| Social Influence | 0.580 | 0.762 | 0.467 (with Intention of Use) | ☑ | Good discriminant and convergent validity |
| Hedonic Motivation | 0.806 | 0.898 | 0.707 (with Emotion) | ☑ | Excellent discriminant and convergent validity |
| Anthropomorphism | 0.805 | 0.897 | 0.294 (with Effort Expectancy) | ☑ | Excellent discriminant and convergent validity |
| Performance Expectancy | 0.659 | 0.811 | 0.456 (with Intention of Use) | ☑ | Good discriminant and convergent validity |
| Effort Expectancy | 0.732 | 0.856 | 0.348 (with Objection) | ☑ | Excellent discriminant and convergent validity |
| Emotion | 0.692 | 0.832 | 0.707 (with Hedonic Motivation) | ☑ | Good discriminant and convergent validity |
| Intention of Use | 0.798 | 0.893 | 0.641 (with Hedonic Motivation) | ☑ | Excellent discriminant and convergent validity |
| Objection | 0.657 | 0.811 | 0.538 (with Intention of Use) | ☑ | Good discriminant and convergent validity |

Note. √AVE values (in column 3) were compared with the highest inter-factor correlation (column 4). Discriminant validity is supported when √AVE exceeds the strongest latent correlation for each construct [29]. All subscales met the criterion.

* Thresholds: AVE ≥ 0.50 = good convergent validity; √AVE > inter-factor correlations = good discriminant validity.
